# Mapping Ethnic Stereotypes and Their Antecedents in Russia: The Stereotype Content Model

**DOI:** 10.3389/fpsyg.2019.01643

**Published:** 2019-07-16

**Authors:** Dmitry Grigoryev, Susan T. Fiske, Anastasia Batkhina

**Affiliations:** ^1^National Research University Higher School of Economics, Moscow, Russia; ^2^Princeton University, Princeton, NJ, United States

**Keywords:** stereotype content model, ethnic stereotypes, cultural distance, intergroup threat, network analysis, differentiated threat

## Abstract

The stereotype content model (SCM), originating in the United States and generalized across nearly 50 countries, has yet to address ethnic relations in one of the world’s most influential nations. Russia and the United States are somewhat alike (large, powerful, immigrant-receiving), but differ in other ways relevant to intergroup images (culture, religions, ideology, and history). Russian ethnic stereotypes are understudied, but significant for theoretical breadth and practical politics. This research tested the SCM on ethnic stereotypes in a Russian sample (*N* = 1115). Study 1 (*N* = 438) produced an SCM map of the sixty most numerous domestic ethnic groups (both ethnic minorities and immigrants). Four clusters occupied the SCM warmth-by-competence space. Study 2 (*N* = 677) compared approaches to ethnic stereotypes in terms of status and competition, cultural distance, perceived region, and four intergroup threats. Using the same Study 1 groups, the Russian SCM map showed correlated warmth and competence, with few ambivalent stereotypes. As the SCM predicts, status predicted competence, and competition negatively predicted warmth. Beyond the SCM, status and property threat both were robust antecedents for both competence and warmth for all groups. Besides competition, cultural distance also negatively predicted warmth for all groups. The role of the other antecedents, as expected, varied from group to group. To examine relative impact, a network analysis demonstrated that status, competition, and property threat centrally influence many other variables in the networks. The SCM, along with antecedents from other models, describes Russian ethnic-group images. This research contributes: (1) a comparison of established approaches to ethnic stereotypes (from acculturation and intergroup relations) showing the stability of the main SCM predictions; (2) network structures of the multivariate dependencies of the considered variables; (3) systematically cataloged images of ethnic groups in Russia for further comparisons, illuminating the Russian historical, societal, and interethnic context.

## Introduction

When people are making sense of individuals or groups, they turn to two basic dimensions in social cognition (Abele et al., under review). The stereotype content model (SCM) terms these dimensions perceived warmth (morality and sociability) and competence (ability and assertiveness), which reflect two general questions about others: “Do they intend to help or harm me (i.e., they are friend or foe); and are they capable of it?” (Fiske et al., 2007; Fiske, 2015, 2018). The SCM has proved robust across cultures and contexts (see e.g., Cuddy et al., 2008, 2009; Fiske and Durante, 2016; Fiske, 2017).

The SCM particularly provides evidence for ethnic stereotypes as accidents of history. In different countries, the four combinations of warmth and competence^1^ include different ethnic groups, depending on social context, national history, and immigration circumstances (Fiske, 2018). These combinations of warmth and competence are accompanied by distinct emotions and behavioral tendencies (Cuddy et al., 2008; Fiske, 2018).^2^

From the outset, the SCM suggested that the main antecedents of the stereotype content are status (societal resources and prestige) and competition (incompatibility of outgroup goals with those of the ingroup) (Fiske et al., 2002). Later, using the Integrated Threats Theory (Stephan and Stephan, 2000), the SCM view on competition incorporated both tangible and symbolic threats for measuring competition (see Kervyn et al., 2015). This line continues here considering additional antecedents of stereotype content.

We also develop the SCM by exploring a new context. Like many psychological theories, the SCM comes from the United States and is most studied there. Despite its size, influence, and host status, Russia has been understudied (Grigoryev and van de Vijver, 2018; Grigoryan, 2019). No articles published have yet used the SCM to examine Russian ethnic stereotypes. In its intergroup relations, Russia is distinctive among other countries. The Russian population is diverse. According to the Census Data for 2010, although ethnic Russians constitute 81% of the population^3^, but the index of ethnocultural diversity in some Russian regions and cities is high and growing (Safronov, 2015). The Russian Federation is historically a plural society, comprising more than 190 ethnic groups, and the territory of the Russian Federation includes 21 national republics. Ethnic Russians are an ethnic minority in some national republics of Russia. The United Nations estimated the Russian Federation to be the world’s second-leading country in hosting the most immigrants in 2013, after the United States. After the European refugee crisis in 2015, Russia came into the third place by a small margin (Grigoryev and van de Vijver, 2018).

Moreover, ethnic groups have variable images in Russian society, so they provide a rich test of the SCM warmth and competence dimensions on a non-American, non-European nation. Also, Russia’s resident ethnic groups may differ in two other variables: their cultural distance and contact with ethnic Russians.

### Incompatible Attitudes, Cultural Distance

Consistent with SCM and the integrated threat view, perceiving symbolic incompatibility or large cultural distance links to viewing ethnic groups as alien or threatening (Lebedeva et al., 2017; Grigoryev et al., 2018). Ethnic Russians perceive a gap between their preferred and immigrants’ “actual” acculturation attitudes, leading to intergroup bias and threats (Grigoryev et al., 2018). Moreover, the attitudes of ethnic Russians toward migrants are negative, despite obvious economic need for labor migrants. Also, the term “migrants” mostly implies people from Central Asia and the Caucasus, often considered an economic burden and cultural threat (Lebedeva et al., 2017; Grigoryev et al., 2019). Cultural threat predicts perceived intergroup conflict in Russia (Minescu and Poppe, 2011).

### Limited Contact

Russia as a country has the world’s largest landmass, so direct intergroup contact (or personal experience) between ethnic Russians and many other ethnic groups is also limited. Consistent with Allport’s (1954) contact hypothesis, ethnic Russians often use stereotypes to evaluate other ethnic groups (about contact and the SCM see Kotzur et al., 2018). Also reducing contact, historically established ethnic hierarchies remain typical for post-Soviet Russia (Hagendoorn et al., 1998, see also Bessudnov, 2016). For example, ethnic Russians probably categorize immigrants from Transcaucasia and Central Asia in the same outgroup as internal migrants from Russian regions of the North Caucasus, whereas placing immigrants from Ukraine and Belarus in another group (Grigoryev and van de Vijver, 2018).

### Threats

Under minimal contact, the most likely source of discord is a variety of perceived intergroup threats that also inform the image of the outgroups. The Intergroup Threat Theory divides all intergroup threats into two types: symbolic (intangible: threats to identity/worldview, consistent with social distance) and realistic (tangible: threats to resources/well-being), at group and individual levels (e.g., the threat of oneself being robbed vs. the threat of the economic burden that affects the whole ingroup). Different types of threats have different consequences (e.g., intangible threats are more associated with a moral evaluation of outgroups) (Stephan and Mealy, 2011). Both types of threats are relevant for Russia; for example, migrant workers from former post-Soviet republics with a poor economic situation (tangible threat) are often Muslims with distinguishing values and identity (symbolic threat). Moreover, the perceived structural features of this region (poor, few natural resources) by themselves can be associated with the stereotype content (see Linssen and Hagendoorn, 1994), perhaps through both perceived tangible threat (low-wage labor competition) and symbolic threat (“peasant” class culture).

### SCM Images

As noted, in Russia, ethnic groups present a rich variety of images, not yet systematically cataloged. The SCM approach predicts that societal groups will spread across the four quadrants, especially in countries with high income-inequality (Durante et al., 2013) and moderate peace-conflict (Durante et al., 2017). Compared with other nations studied so far, Russia’s Gini coefficient of income inequality in 2015 is 41.2, which indicates a moderate level of inequality (not as unequal as South Africa, nor as equal as Sweden), predicting some ambivalent stereotypes to appear. But Russia’s GPI in 2018 is 3.16, which is an extremely high value on conflict.^4^ So we might also expect predominantly unambivalent stereotypes.

Russia potentially challenges the SCM. If compared with the United States, where the SCM has been most often studied, both countries have moderate inequality; also, both are among the five most militarized countries in the world. However, Russia scores poorly on GPI while the United States scores moderately. In addition, the World Value Survey shows that Russia and the United States are in opposite quadrants of traditional vs. secular-rational and survival vs. self-expression values dimensions. Moreover, Russia had for some time shown the reverse of the global trend and moved to the values of survival and still continues moving toward more traditional values (Inglehart, 2018). In addition, Russia and the United States differ considerably on all of the six Hofstede dimensions (Hofstede et al., 2010).^5^ So the United States and Russia, while having some similar global positions, have different development, history, and culture. Thus, such a distinct context as Russia can contribute to better understanding universal and culture-specific patterns in the SCM framework.

This paper respects the Russian cultural context and tests the SCM’s fit. The SCM could be falsified, as not a good fit, if Russian ethnic groups fail at most of the following: (a) spread out across the warmth-by-competence space; (b) form multiple (3−5) clusters; (c) have one or more ambivalent (mixed) clusters; (d) follow structural antecedents of stereotypes; (e) show ambivalence (or not) as a function of national indicators; and (f) predict downstream consequences. This article tests all criteria but the last.

An emic critique overlaps with other critiques of the SCM’s theory-driven approach (e.g., Koch et al., 2016) in determining which stereotype content to test. However, more data-driven, open-ended responses in several settings still reproduce the SCM dimensions (Nicolas et al., under review), especially when perceivers have a relational goal (Nicolas et al., under review).

Finally, the emic and data-driven approaches might critique standard SCM methods for pre-determining which groups to rate (i.e., although supplied by open-ended listing, then choosing the most frequent mentions, the instructions do include prompts^6^). One response is to use a culture’s official list, designed for another purpose, and therefore not subject to researcher bias; the current project follows that strategy by using government lists; an earlier study used EU member nations for the same reason (Cuddy et al., 2008, 2009). Another response is a broader, even less constrained list (Nicolas et al., under review). Indeed, a simultaneous project on social class stereotypes, in eight post-communist countries, documents SCM responses to spontaneously mentioned Russian social groups, instead of using the official list of ethnic groups, as done here (Grigoryan et al., under review). Those data meet the SCM criteria (a–e, described above).

### Overview

This research includes two parts. The first study determined the Russian SCM map of ethnic stereotypes, tested some of the assumptions from the SCM and other relevant intergroup frameworks, and selected prototype groups for the second part of the research. Specifically, we expected a replication of apparently universal patterns locating ethnic groups along the two SCM dimensions, multiple clusters, some ambivalent, and correlated with structural antecedents; as well, we expected both dimensions to favor cultural/religious similarity to native-born ethnic groups.

The second study explored various approaches to predicting ethnic stereotypes, addressing social structure (SCM; e.g., Fiske, 2018), perceived cultural distance (e.g., Suanet and van de Vijver, 2009), perceived structural features of a region (e.g., Linssen and Hagendoorn, 1994; Kotzur et al., 2019), and intergroup threats (e.g., Cottrell and Neuberg, 2005; Stephan and Mealy, 2011). So besides status and competition as the main SCM antecedents of ethnic stereotype content, we extended the previous framework of cultural similarity and added newly conceived socioecology (Linssen and Hagendoorn, 1994) and sociofunctional threats (Cottrell and Neuberg, 2005) perspectives. We expected a replication of basic SCM hypotheses, even in competition with the other explanations. To explore the relative roles of the various predictors (i.e., to get a detailed story of the multivariate dependencies in these data), network analysis estimated and graphed their network structures.

Thus, this research makes several contributions: (1) a comparison of various established approaches to ethnic stereotypes from the acculturation literature and intergroup relations showing the stability the main of predictions of the SCM; (2) network structures of the multivariate dependencies of the considered variables; (3) systematically cataloged images of ethnic groups in Russia for further comparative perspectives.

## Study 1: Mapping Ethnic Stereotypes

The aim of this study was generating the Russian SCM map in order to understand how different ethnic groups living in Russia spread across the SCM space. Moreover, we seek to determine prototype groups for further research on Russia in the SCM framework. Specific ethnic groups can differentially locate at various points along the two SCM dimensions (Lee and Fiske, 2006; Vaes and Paladino, 2010; Sibley et al., 2011; Binggeli et al., 2014; Kotzur et al., 2019). In this exploratory study, we expected that perceptions of specific ethnic groups would vary, such that the groups occupy distinct locations in the SCM space, with some groups receiving ambivalent stereotypes:

H1 and H2. Some universal patterns emerge across countries (see e.g., Cuddy et al., 2008, 2009; Bye et al., 2014; Asbrock, 2010; Janssens et al., 2015; Fiske and Durante, 2016; Fiske, 2017; Stanciu et al., 2017; Grigoryan et al., under review); these should also characterize ethnic Russian perceivers in Russia. For instance (H1a), the Russian ingroup and historic allies (e.g., Belarusians and Ukrainians) should locate in a cluster corresponding to high-competence and high-warmth. (H1b) Jews, East Asians (e.g., Japanese and Chinese), and developed Western countries (e.g., German and English) should locate in a cluster corresponding to high-competence and low-warmth. (H1c) Some indigenous groups (e.g., Buryats and Udmurts) should locate in a cluster corresponding to low-competence and high-warmth. (H1d) Finally, stigmatized groups (e.g., Roma and Chechens) and groups associated with immigrants who have low-skilled jobs (e.g., Uzbeks and Tajiks) should locate in a cluster corresponding to low-competence and low-warmth. In addition, (H2) Russia’s high-conflict GPI suggests few ambivalent (mixed) stereotypes, so predominantly unambivalent stereotypes are expected.

H3. Cultural distance, as noted, can have substantial consequences for intercultural relations (Galchenko and van de Vijver, 2007; Ward and Geeraert, 2016; Grigoryev et al., 2018), so also in Russia (e.g., Suanet and van de Vijver, 2009). Russia is not only a polycultural but also multidenominational country, and religion for ethnic Russians can bind ethnic groups more than territorial proximity and economic cooperation. Considering religion as a proxy for cultural distance (or addressing religious distance directly) can use Census Data (see Supplementary Table S1) to classify the ethnic groups by religious denominations; ethnic Russians can have different evaluations for Christian groups and the representatives of other religions (see also Grigoryan, 2019; also see Kotzur et al., 2019). Because ethnic Russians are mainly Eastern Orthodox Christians, this denomination has had a strong influence on Russian culture. Moreover, they are sufficiently likely to know the religious affiliation of other ethnic groups (Balzer, 2015; Grigoryev et al., 2018). So (H3), religious differences can be a source of antipathy because of perceived group dissimilarity (in values, beliefs, attitudes; Stephan and Stephan, 2000; Costa-Lopes et al., 2012).

H4 and H5. The classic dichotomy of ingroup and outgroup (e.g., Hewstone et al., 2002) may manifest in ethnic Russians’ evaluations of other ethnic groups. In general: (H4) indigenous ethnic groups that live in the Russian Federation as native-born should have higher evaluations than other ethnic groups (e.g., Tatars, Chuvashs, Kalmyks, Dargins, Yakuts, etc., vs. Poles, Arabs, Bulgarians, Spanish, Japanese, etc.). Also, because of increased contact, (H5) larger groups should have higher evaluations than smaller groups.

## Method

### Participants

The total sample of 438 ethnic Russian participants included 46.8% women and 53.2% men, aged from 16 to 69 (*M* = 30.5, *SD* = 10.8); 69.4% had a university education; 53.7% were Russian Orthodox Christians [other participants had not affiliated with any religion (i.e., secular or atheists)], and 25.3% were students.

### Procedure

The data were collected online via social media in 2018. All participants completed the questionnaire voluntarily and did not receive any remuneration. We recruited participants using targeted, paid ads in “VK”, the most popular social network in Russia. This social network covers more than 90 million Russian citizens, which provides good access to major parts of the Russian population. Participants received the instructions, which included information about the main topic discussed in the study (there is a diversity of ethnic groups in Russia but still little is known how ethnic Russians perceive these groups), confidentiality policy, and how to contact the researchers. The informed consent of the participants was implied through survey completion.^7^

To compose the questionnaire, as the first step, we selected 60 ethnic groups based on their numbers in Russia, according to 2010 census data and 2017 data from the Main Directorate on the Issues of Migration of the Ministry of Internal Affairs of Russia because some ethnic groups are more immigrant groups (e.g., number of Japanese 835 as citizens and 75,148 as immigrants, number of Chinese 28,943 as citizens and 1,506,110 as immigrants, etc.). The information appears in the, Supplementary Tables S1, S2. The next step randomly split these groups into three subsets (20 ethnic groups per set) to randomly present one subset for each participant to evaluate. This split reduced participants’ cognitive load and facilitated their completing the questionnaire.

### Measures

All measures were administered in Russian. The questionnaire contained the translated measures, shaped by back-translation and cognitive interviews with the think-aloud technique (Willis, 2004). In the questionnaire, participants rated each target group from their subset of 20, on perceived warmth and competence, using a 5-point Likert scale (1 = *not at all*; 5 = *extremely*), according to the SCM instructions, which asks how they think their society views each group (Fiske et al., 2002). The SCM measure covered three warmth items (sincere, trustworthy, warm) and three competence items (effective, capable, competent). In each group, both scales showed satisfactory reliability, with Cronbach’s alpha ranging from 0.70 to 0.92 (overall means of 0.84 and 0.84 for both competence and warmth).

We additionally asked participants, for further selection of prototype ethnic groups that are easier to evaluate:

If it was difficult for you to provide your opinion on the opinion of the majority of ethnic Russians about the above groups, then indicate these groups in the list below. If such difficulties have not arisen, then skip this question. (You can choose several answers at once).

Finally, the questionnaire also included sociodemographic variables [gender, age, religious affiliation, level of education, and student status (yes/no)] and a self-reported attention check at the end to exclude low-quality answers. The self-reported attention check contained one question:

Sometimes people are able to concentrate completely on answering a survey, and sometimes people cannot give very much attention. Please tell us honestly how much attention you gave to the survey. (1 = *very little attention*; 7 = *complete attention*).

### Data Analysis

Data screening included checking for outliers and missing data. Also, for the self-reported attention check scale, we established the threshold of two or less to exclude cases. For the preliminary analysis, we used Finn’s coefficient of interrater agreement, Cronbach’s alpha for internal consistency, correlation analysis, paired sample *t*-test, and analysis of variance (ANOVA) (with *post hoc* tests used Tukey’s multiple testing correction).

Further, according to the SCM clustering procedure (Cuddy et al., 2009), two cluster analyses determined, respectively, first, the number of clusters and then their membership. A first hierarchical cluster analysis used Ward’s method (minimizing within-cluster variance and maximizing between-cluster variance). On the next step, *k*-means cluster analysis using the parallel threshold method revealed the cluster membership of each target group.

## Results

### Preliminary Analysis

The data had no outliers or missing values. The self-reported attention check scale ranged from 3 to 7 (*M* = 5.86, *SD* = 0.86), so the data had no observations that were consistent with our criterion for exclusion.

The main information including means, standard deviations, internal consistency, interrater agreement can be found in Supplementary Table S3. In each group, competence and warmth scales (three items each) showed Cronbach’s alpha ranging from 0.70 to 0.92 (overall means of 0.84 for both), showing acceptable internal consistency for each measure. Interrater agreement for evaluations of the target groups ranged from 0.68 to 0.88 (*M* = 0.79, *SD* = 0.04) for competence and from 0.63 to 0.86 (*M* = 0.76, *SD* = 0.05) for warmth, which indicates that ethnic Russians perceived these groups with sufficient agreement (homogeneity).

For ethnic Russians, reporting the opinion of most ethnic Russians was hardest for Tuvans, Argentines, Udmurts, Hungarians, and Komi. In contrast, Chechens, Armenians, Germans, ethnic Russians, and Georgians were reportedly easier to rate. Larger size accompanied reporting an easier evaluation of the target groups by ethnic Russians. That is, mentioning groups as difficult to evaluate was negatively correlated with the number of immigrants according to 2017 statistics (*r* = −0.35, *p* = 0.029).

### Cluster Analysis

**FIGURE 1:**
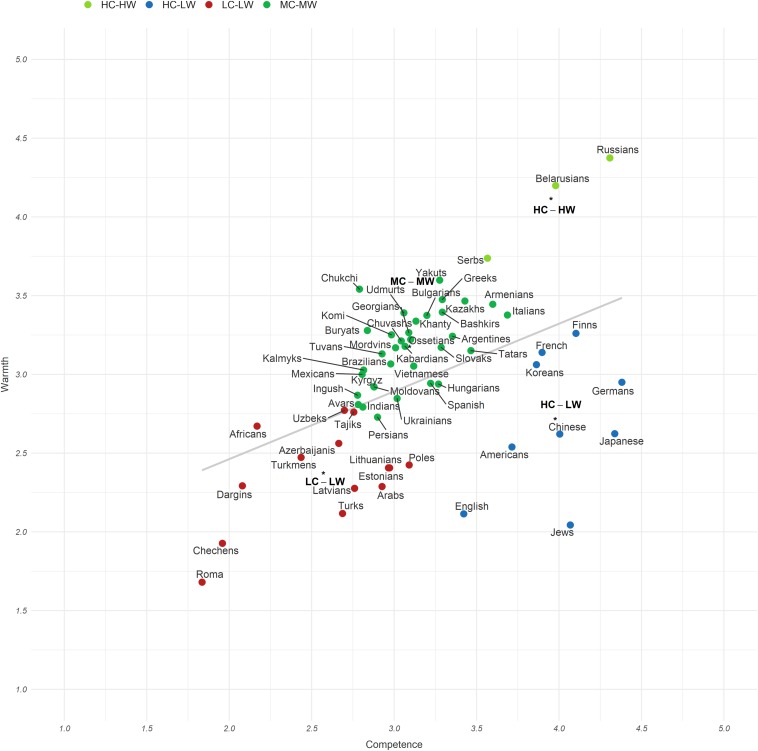
The Russian SCM map of ethnic stereotypes.

The hierarchical cluster revealed agglomeration statistics that supported a four-cluster solution. The information about cluster membership appears in Supplementary Table S3. The distribution of the ethnic groups on the SCM map appears in Figure 1. We found four clusters: high competence and high warmth (HC-HW), middle competence and middle warmth (MC-MW), high competence and low warmth (HC-LW), and low competence and low warmth (LC-LW). The paired sample *t*-test comparisons of competence and warmth means for each cluster appear in Table 1. Based on the cluster results, most groups are unambivalent (fitting prediction H2), a few are HC-LW ambivalent (fitting prediction H1b), and none are LC-HW ambivalent (contrary to prediction H1c).

**TABLE 1:** Competence and Warmth means for each cluster, study 1.

	***M* (*SD*)**			***t*(df)**	***d***
	**Competence**		**Warmth**		
HC-HW	3.95 (0.37)	=	4.10 (0.33)	−3.370(2)	0.37
MC-MW	3.09 (0.24)	=	3.16 (0.23)	−1.798(33)	0.30
LC-LW	2.57 (0.41)	=	2.36 (0.31)	2.137(13)	0.57
HC-LW	3.98 (0.30)	>	2.71 (0.44)	8.861(8)^*^	3.35

^*^p < 0.001.

### Ambivalent Stereotypes

Also testing the prediction (H2) that Russia would have predominantly unambivalent stereotypes, out of 60 groups rated, only 16 can be classified as ambivalent (mean difference showed medium effect size, *d* = 0.50) (see Supplementary Table S3). Nine groups (Jews, Japanese, Germans, Chinese, English, Americans, Finns, Koreans, and French) were rated significantly more competent than warm with large effect size, *d* = 0.80 (prediction H1b): five high-competence groups showed the highest difference between the competence and warmth dimensions: Jews (*M*_*diff.*_ = 2.03, *p* < 0.001, *d* = 1.88), Japanese (*M*_*diff.*_ = 1.72, *p* < 0.001, *d* = 1.84), Germans (*M*_*diff.*_ = 1.43, *p* < 0.001, *d* = 1.62), Chinese (*M*_*diff.*_ = 1.38, *p* < 0.001, *d* = 1.48), English (*M*_*diff.*_ = 1.31, *p* < 0.001, *d* = 1.24). Although smaller in absolute magnitude, five high-warmth groups had the highest difference between the competence and warmth dimensions (prediction H1c): Chukchi (*M*_*diff.*_ = −0.75, *p* < 0.001, *d* = 0.78), Africans (*M*_*diff.*_ = −0.50, *p* < 0.001, *d* = 0.52), Buryats (*M*_*diff.*_ = −0.44, *p* < 0.001, *d* = 0.48), Udmurts (*M*_*diff.*_ = −0.33, *p* < 0.001, *d* = 0.35), Yakuts (*M*_*diff.*_ = −0.32, *p* < 0.001, *d* = 0.34). One ambivalence criterion is a significant difference and its effect size, as here. Another is cluster membership (above), and a third is the warmth-competence correlation (see those analyses next). None indicates any HW-LC groups, and the vast majority of groups are rated unambivalently.

### Correlation Between Competence and Warmth Dimensions

As mentioned, Russia’s Gini coefficient of income inequality and GPI, respectively, show moderate inequality but really high conflict. In accord with the GPI score, the competence and warmth dimensions correlated positively (*r*(58) = 0.46, 95% *CI* = [0.23, 0.64], *p* < 0.001) (H2). In other SCM samples (see Durante et al., 2017), the warmth-competence correlation ranges from −0.19 to 0.92, moderated by variables such as inequality and peace-conflict, as mentioned, so the Russian sample is in the middle of the SCM range and moderate for an effect size. Furthermore, visual inspection of the corresponding figure looks less like a vector than a large lump with two ingroup outliers (Russians and Belarusians) at the high-high end and two extreme outgroups (Chechens and Roma) at the low-low end. Removing these outliers lowers the correlation to *r*(54) = 0.20, 95% *CI* = [−0.11, 0.49], *p* = 0.149, suggesting that the outliers are responsible for the high correlation. The difficulty in evaluating an ethnic group does not qualify the overall warmth-competence ambivalence; the partial correlation controlling for the difficulty in evaluating an ethnic group is *r*_*p*_(57) = 0.54, 95% *CI* = [0.29, 0.72], *p* < 0.001.

The mixed stereotypes had a small number of ethnic groups from economically developed countries (e.g., United States, German, France, Japan, China, etc.) that were located in the HC-LW cluster, consistent with prediction (H1b). The other ethnic groups traced the strict diagonal hierarchy of unambivalent stereotypes, also fitting predictions (H1a, H1d, and H4): from Orthodox Slavic peoples (HC-HW) to socially excluded groups and conflicting groups (LC-LW). The mainly indigenous peoples of Russia and Eastern Christian groups locate in the MC-MW quadrant, while the LC-HW quadrant did not appear at all, contrary to prediction (H1c).

### Size and Ingroup/Outgroup Positions

Moreover, as our prediction (H5) proposes, the size of target groups according to the Census data was positively correlated with the evaluation on both the competence dimension (*r* = 0.28, *p* = 0.033) and warmth dimension (*r* = 0.37, *p* = 0.004), as well as the interrater agreement for each of them: competence (*r* = 0.32, *p* = 0.014) and warmth (*r* = 0.27, *p* = 0.035), respectively. The predicted pattern appeared, such that large ethnic groups were evaluated more positively and consistently. Furthermore, the ethnic groups that live in the Russian Federation as native-born scored higher on warmth (*M* = 3.17, *SD* = 0.50) than other ethnic groups (*M* = 2.86, *SD* = 0.50), *F*(1, 58) = 4.963, *p* = 0.030, ω^2^ = 0.062—but not on competence, *F*(1, 58) = 2.470, *p* = 0.121, ω^2^ = 0.024.

### Religious Denominations and the Stereotypes

Furthermore, consistent with prediction (H3) on cultural distance, a group’s religion had consequences for warmth [*F*(3, 56) = 5.173, *p* = 0.003, ω^2^ = 0.173] and competence [*F*(3, 56) = 3.564, *p* = 0.020, ω^2^ = 0.114]. Islamic groups (*M* = 2.80, *SD* = 0.42) were evaluated as less competent than Western Christian groups (*M* = 3.37, *SD* = 0.47), *M*_*diff.*_ = 0.57, *SE* = 0.18, *t* = 3.105, *p* = 0.015, *d* = 1.28). Eastern Christian groups (*M* = 3.33, *SD* = 0.58) were also evaluated as warmer than Western Christian groups (*M* = 2.83, *SD* = 0.40) and Islamic groups (*M* = 2.74, *SD* = 0.44), respectively, *M*_*diff.*_ = 0.50, *SE* = 0.16, *t* = 3.035, *p* = 0.019, *d* = 0.99 and *M*_*diff.*_ = 0.59, *SE* = 0.16, *t* = 3.629, *p* = 0.003, *d* = 1.15.

## Discussion

Testing eight predictions (H1a−H5), this study addressed the positions of Russian ethnic groups on the SCM map and their cluster memberships. Four clusters emerged, with the following membership: Orthodox Slavic peoples (HC-HW), mainly indigenous peoples of Russia and Eastern Christian groups (MC-MW), socially excluded groups and conflict groups (LC-LW), and ethnic groups from economically developed countries (HC-LW). In addition, we tested our assumptions about the ambivalence of stereotypes, the correlation between competence and warmth dimensions, sizes, in- and out-group positions, and cultural or religious similarity.

### Ambivalence

Ambivalence of stereotypes (i.e., low correlation between warmth and competence) can reflect the macro-social conditions in a country (Fiske and Durante, 2016), such as social inequality (Durante et al., 2013) and peace-conflict (Durante et al., 2017). The moderate positive correlation between warmth and competence indicated low stereotype ambivalence, in comparison to the United States, but not so high as to make the two dimensions are not redundant. The moderately high correlation fits Russia’s GPI better than Russia’s Gini coefficient (H2). Moreover, SCM work on Global Peace-Conflict indicates that Russia’s GPI is as high as Pakistan, close to Iraq and approaching Afghanistan, all of which have moderate-to-high warmth-competence correlations, so Russia’s warmth-competence correlation fits its profile. The level of interethnic tension in Russia is still high (Minescu and Poppe, 2011), which demands stricter divisions between the preferred groups and predominantly unambivalent negative stereotypes, “Us vs. Them.”

### Clusters

As noted, predictions assumed that four clusters would be distinguished by the following characteristics: (H1a) ingroup and allies (HC-HW), (H1b) Jews, East Asians, and developed Western countries (HC-LW), (H1c) some indigenous groups (LC-HW), and (H1d) stigmatized groups and perceived as unskilled immigrants (LC-LW). The *majority* of the data points vary among themselves in ways that resemble other SCM plots: successful foreigners in the HC-LW quadrant (e.g., Chinese, Germans, and Finns); Africans and Turks in the LC-LW quadrant; Italians in the LC-HW (or MC-MW) quadrant; and tolerated allies (Serbs) in the HC-HW quadrant (see Bergsieker et al., 2012, Study 4; Lee and Fiske, 2006; Cuddy et al., 2009).

#### HC-HW

Only three groups entered the most highly valued cluster: Russians (auto-stereotypes), Belarusians, and Serbs; this cluster thus contained Orthodox Slavic peoples (so-called in Russia “fraternal peoples”). The exception was Ukrainians (MC-MW). Although some time ago Ukrainians were evaluated highly (see Hagendoorn et al., 1998), the crisis in Russian-Ukrainian relations and the growing negative discourse about Ukraine and Ukrainians in the media could have led to this dramatic change.

As a rule, individualistic countries tend to evaluate themselves as more competent, whereas collectivist ones tend to be more warm (Cuddy et al., 2009). At the same time, Russians rated themselves extremely highly (see also Allik et al., 2010), in both competence and warmth; this may be because, in post-Soviet Russia, the values transformation is still going on, and at the moment Russian society is located at the borderline between individualistic and collectivistic countries (Minescu et al., 2008). In addition, the trend laid down in the Soviet Union may persist when, despite the policy of maintaining cultural diversity, Russians perceive themselves at the highest position in the national hierarchy (Tishkov, 1994).

#### LC-HW

The assumption (H1c) about indigenous groups was not borne out: they located in the MC-MW cluster, while the LC-HW cluster did not appear at all. From one side, a different clustering from Western countries is normal in terms of cultural variation (Fiske and Durante, 2016), e.g., in Eastern countries differing cluster solutions were also obtained in a few studies. It is also normal because hardly any nation locates ethnic groups in the LC-HW quadrant (pity quadrant, see Cuddy et al., 2008), which more often correspond to specific social groups such as children, old people, and disabled people.

#### MC-MW

The indigenous peoples of Russia (e.g., Chukchi, Komi, and Buryats) were evaluated as warmer indeed but less competent than the representatives of some foreign countries (e.g., Italians, Slovak, and Spanish). This fits mundane attitudes of ethnic Russians toward certain indigenous peoples (Tishkov, 1994), e.g., in Russia, widespread jokes about the Chukchi represent them as narrow-minded. We can see that in the cluster MC-MW ethnic groups that have seemingly nothing in common can be found. Thus, one explanation may be that these social categories were not sufficiently salient in the minds of participants, and as a result their answers were neutral overall. The similar results appear in the United States: consistent with for example neutral ratings of Native Americans in the Northeast, where they are not salient (Fiske et al., 2002), versus differentiated in the Southwest, where they are salient (Burkley et al., 2017).

#### LC-LW

As expected, socially excluded groups and conflict groups located in the LC-LW cluster. Many of these groups (e.g., Uzbeks, Tajiks, and Azerbaijanis) also have a predominantly negative image in the Russian media (Khaptsova et al., 2018). In addition, in a recent SCM study that included Russian social groups, the groups that primarily refer migrants, Muslims, and Caucasians also located in the LC-LW cluster (Grigoryan et al., under review; see also Bessudnov, 2016). Inhabitants of Baltic countries and Poles were among the worst evaluated cluster, although they are neither stigmatized nor illegal immigrants, traditionally estimated to be the lowest (Durante et al., 2013). This low-low stereotyping may relate to the problems of Russia’s relations with the former post-Soviet republics and the countries of the socialist bloc, as well as to the intergroup polarization that occurred after the collapse of the Soviet Union (Hagendoorn et al., 1998). As in the Ukraine case, similar consequences for the low evaluation of Turks (LC-LW) could have been the recent conflict with Turkey, despite Russian media for some time reporting about exclusively positive intergroup contact with Turks (Khaptsova et al., 2018).

#### HC-LW

Several ethnic groups from economically developed countries had mixed HC-LW stereotypes. These envy-quadrant stereotypes combine competence with coldness, portraying these ethnic groups as powerful, self-serving competitors, with negative intentions toward ethnic Russians (Cuddy et al., 2008). When the warmth-competence correlations are moderate to high, as in Russia, the correlations indicate that the distribution of groups in the SCM space tends toward a vector, instead of a cloud of points. The vector minimizes the ambivalent clusters, almost always the ambivalent cluster that disappears is the pity quadrant, but the resented envy quadrant usually stays HC-LW, as it does here (Durante et al., 2017).

### Distance

Our assumption (H4) regarding group similarity and closeness (religious affiliation or cultural distance, and the dichotomy of the ingroup and outgroup) was supported. Islamic groups were evaluated lower than Western Christian groups, and Western Christians were evaluated lower than Eastern Christian groups. Other countries also rate the representatives of their own religious group as more competent and warmer than the others (Fiske and Durante, 2016; Fiske, 2017). This result also corresponds to patterns for perceived similarity in multiple categorization in Russia (see Grigoryan, 2019).

According to van Osch and Breugelmans (2012), perceived intergroup difference, which extends the distinction that people make between ingroups and outgroups, is a prime candidate for organizing attitudes among various groups in culturally diverse societies. Groups differ in their societal position—as defined by the consensual perceived differences among groups by the society members. Those non-dominant groups who were perceived as less different from dominant group members “received simultaneously more support for multiculturalism; were seen as less threatening, more warm and more competent; were preferred to adopt less and maintain more; but perceived to adopt more and maintain less” (p. 10). This fits the literature on ethnic hierarchy, which support the assumption of strong consensus among groups with regard to intergroup differences. An ethnic hierarchy implies a societal rank order of groups on the basis of perceived differences among groups (see Hagendoorn, 1995). Locations in the social structure affect observations of intergroup relations, and group members’ typical roles shape stereotype content (Koenig and Eagly, 2019); this societal rank order fits the perceived social structure.

Potential moderators for the acculturation process include (Brown and Zagefka, 2011): (1) conceptualizations of nationality and ethnicity that prevail in given societal contexts [especially their (non)essentialist character]; (2) magnitude of the perceived cultural difference between the majority and minority cultures; and (3) life domain of the acculturation preferences. Perhaps all of them can reduce to the issue of perceived group similarity (or in general the dichotomy of the ingroup and outgroup): (1) perceived cultural difference is just one of the aspects of perceived group similarity; (2) conceptualization of nationality and ethnicity is an issue about group borders—borders are more permeable for more similar groups; (3) domain-specificity just separates the cultural difference on several particular domains—that is, specifies perceived cultural difference and similarity in various aspects (e.g., family relations, consumer habits, clothes, etc.). Thus, these phenomena connect the acculturation and intergroup relations literatures.

### Selection of Prototypic Groups for Study 2

Based on a combination of information about distances of cluster centers, difference between competence-warmth dimensions, size of groups (number according to the Census), interrater agreement, and respondent rated difficulties in assessing^8^, we selected five prototype groups: Belarusians (HC-HW), Armenians (MC-MW), Buryats (LC-HW), Chechens (LC-LW), and Chinese (HC-LW).

## Study 2: Comparing Approaches

Study 1 showed only partial support for SCM predictions, so this study searched farther for complementary approaches to explain the Russian patterns. This study combined different approaches to ethnic stereotypes with the SCM framework to assess their comparative outlook. The SCM posits that social structure mainly explains variations in the warmth-by-competence space; this social structure includes respective antecedents: (a) perceived status predicts perceived competence, while (b) perceived interdependence (competition/cooperation) predicts perceived warmth (Fiske, 2015, 2018). The status-competence correlation is robust (averaging above 0.80), and the interdependence-warmth correlation can reach comparable levels, if the respective variables are measured appropriately (Kervyn et al., 2015).

Nevertheless, social structure is not the only possible antecedent; for example, some studies have addressed other social and geographical factors (Linssen and Hagendoorn, 1994; Poppe, 2001; Kotzur et al., 2019; see also Oishi and Graham, 2010). Moreover, the field of intergroup relations can benefit from acculturation approaches (Ward et al., 2017). In this area, cultural distance is one of the key factors of interethnic relations (Ward and Geeraert, 2016), and proved useful in Study 1. So a combination of research streams can enrich both acculturation research (e.g., adding social structure when considering interethnic relations) and intergroup relations (e.g., adding different cultural distances). From some receiving populations’ perspective, not race (African, Asian, and European) but immigrants’ acculturation strategy (assimilation, integration, separation, marginalization) influences their perceived competence and warmth (Alcott and Watt, 2017). In other settings, race remains important (Lee and Fiske, 2006). Consequently, the SCM antecedents and cultural distance variables might overlap or contribute separately to predict ethnic stereotypes, as Study 1 suggests.

Based on Study 1 criteria, we used five target ethnic groups in the study: Belarusians (HC-HW), Armenians (MC-MW), Buryats (LC-HW), Chechens (LC-LW), and Chinese (HC-LW). We hypothesized following:

H6. Considering social structure to explain stereotypes in the SCM framework (Fiske, 2018), status and competition will explain variations of ethnic stereotypes in their content, that is, status should positively associate with competence (H6a), and competition should negatively associate with warmth across all the ethnic groups (H6b).

H7. In the process of perceiving groups, some global estimate of evaluation may co-exist with the SCM dimensions (see, e.g., Kervyn et al., 2013; Sayans-Jiménez et al., 2017). Taking into account the results of Study 1, perceived cultural distance might work as such a global evaluation in the case of interethnic relations and might show, for example, the degree of similarity between an ingroup and outgroup; majority group members can attribute lower warmth and competence to groups that differ more from them (van Osch and Breugelmans, 2012). So culturally close outgroups, in general, might be evaluated more positively; this fits Taylor’s (1991) argument that similarity promotes attraction (but see also Costa-Lopes et al., 2012). More culturally distant groups may be evaluated less positively on warmth and competence dimensions; that is, perceived cultural distance will be negatively associated with competence and warmth across all the ethnic groups (H7).

H8. The perceived structural features of a region, such as socially and economically (un)favorable conditions, may translate into stereotypical traits of the whole population (e.g., Linssen and Hagendoorn, 1994; Poppe, 2001; see also McCrae et al., 2007). For example, specific ecology can elicit stereotypes (Williams et al., 2016). Sometimes people can imagine the social and economic situation in an ethnic group’s particular region, better than traits of this group. So evaluation of regions as socially and economically unfavorable can negatively associate with the competence of their population (H8).

H9. Competition can cover some threats, and the SCM includes both tangible (resource) and symbolic (value) threats (Kervyn et al., 2015), consistent with the Intergroup Threat Theory (Stephan and Mealy, 2011). However, considering the threats separately, one can form a consistent threat profile for each target group (Cottrell and Neuberg, 2005; Meuleman et al., 2018; Landmann et al., 2019). That is, groups believed to pose qualitatively distinct threats to ingroup resources, values, or processes would elicit qualitatively distinct and functionally relevant reactions.

### Distinct Threats

Four type of threats—physical, property, cultural, and economic—cover the reasonable distinctions: tangible vs. intangible, individual vs. group, and social vs. economic. All these threats represent various hostile intentions, which are negatively associated with warmth of the ethnic groups. The individual-level threats (physical and property) can be more relevant for the warmth of low-competence groups (Buryats and Chechens), who would stereotypically implement these hostile intentions in an unskilled way (H9a). The group-level threats (cultural and economic) must meet two conditions: an ethnic group must be sufficiently numerous (see also Minescu and Poppe, 2011) and competent—conditions met by three ethnic groups in our set: Belarusians, Armenians, and Chinese (H9b).

### Networks

Given all these potential variables, understanding their mutual relations may clarify the overall picture. Finally, thus, the exploratory part of Study 2 investigates network structure of the ethnic stereotypes to better understand common patterns and maybe establish what is central to ethnic stereotypes’ systems. Network analysis, as a multidimensional statistical procedure, makes it possible to clarify the relationships in a set of variables using a combination of special topological logic of the location of variables on a graph and the analysis of pair correlations. In this type of analysis, psychological attributes are a complex system of interactive components (network), i.e., a system in which each component interacts with each other without being tied to a common (unobservable) variable that causes changes (e.g., De Schryver et al., 2015). In this study, network analysis allowed us to construct an easily interpreted and graphically displayed structure of the considered antecedents of ethnic stereotypes in order to better understand the general patterns of interconnections in the network and to establish what is central to the system of considered variables.

Network analysis should reveal the amount and closeness of stereotypes’ associations and also determine which elements are binding (connecting) and confounding (redundant) for other variables. For example, an element with the most direct associations in the network can be considered central. Overall, this analysis should show whether the nodes are isolated or the network contains strong clusters of variables (communities) and the global structure in whole is sparse or dense. The topology, or overall global structural organization, of the ethnic stereotypes and the roles of specific variables in the networks, can emerge in a manner that other statistical approaches cannot provide.

## Method

### Participants

The representative sample of 677 ethnic Russian participants from Central Federal District of Russia (excluding Moscow and Moscow Oblast)^9^ included 55.7% women and 44.3% men, aged from 16 to 79 (*M* = 34.9, *SD* = 11.7).

### Procedure

The data were collected in 2018 by a commercial company that provides paid data collection services for social and marketing research. The company used our questionnaire and their own pool of respondents, who received a compensation of United States $0.75 for their participation. Participants received the same instructions and information as in Study 1.^10^

### Measures

As in Study 1, all measures were administered in Russian. The measures that did not yet have a Russian translation were translated and shaped by back-translation and cognitive interviews with the think-aloud technique (Willis, 2004). In addition, the quality of the new measures and selection of intergroup threats were also informed by a pilot study (*N* = 105) conducted at a university in Moscow. The Study 2 questionnaire contained new test questions to check for respondents’ attention, “If you are reading this, select the option (…).”

#### Antecedent Variables

##### Social structure

Six items with a 5-point Likert scale (1 = *not at all*; 5 = *extremely*) assessed the status and competition (three items per variable) in relation to each ethnic group (Kervyn et al., 2015), with sample items such as “How well educated are (…)?” and “If resources go to (…), to what extent does that take resources away from the rest of society?” Cronbach’s alpha ranged from 0.70 to 0.82 (overall means were 0.75 and 0.76 for status and competition, respectively).

##### Cultural distance

We asked participants to evaluate using a 5-point Likert scale (1 = *not at all*; 5 = *extremely*) differences between Russian and another target culture in ten domains (e.g., beliefs, customs, and traditions; friendship and relationships between people; representations about system of law and legal proceedings; system of education and upbringing; ideals and the meaning of life; art and literature, etc.). A sample of the question for each ethnic group, “In the world there is a great variety of cultures, some of them are very similar and some are quite different. Please evaluate how different Russian culture and (…) culture are in the following domains”. Cronbach’s alpha ranging from 0.85 to 0.97 (overall mean of 0.92).

##### Unfavorable region

Two items with a 7-point Likert scale (1 = *not at all*; 7 = *extremely*) assessed the structural features of a region (socially and economically unfavorable conditions) in relation to each ethnic group, with sample items such as “(…) is a socially unfavorable region,” and “(…)] is an economically unfavorable region”. As regions we considered Belarus, Armenia, Buryatia, Chechnya, and China accordingly. We assume that among Russians, the rated ethnic groups will be directly associated with these regions. Buryatia and Chechnya are national republics within Russia where most Buryats and Chechens living in Russia reside, respectively. Belarus and Armenia are relatively ethnically homogeneous national republics; Belarusians and Armenians will be primarily associated as immigrants coming from these republics. China is a culturally heterogeneous country, but the majority of Russians perceive China citizens as Chinese and, in our opinion, should also determine their belonging to this region. Spearman-Brown coefficient^11^ ranged from 0.66 to 0.84 (overall mean of 0.77).

##### Intergroup threats

Eight items with a 7-point Likert scale (1 = *not at all*; 7 = *extremely*) assessed the physical, property, cultural, and economic threat (two items per variable), in relation to each ethnic group, with sample items such as “(…) threaten the physical security of people like me since they are able to attack for no reason at all”, “(…) threaten the personal stuff of people like me since they are able to steal and/or spoil them”, “(…) threaten the established cultural traditions, customs, and norms of behavior of people like me”, and “(…) threaten the economic welfare of people like me in the labor, housing, and services market”. Spearman-Brown coefficient ranged from 0.58 to 0.94 (overall means of 0.83, 0.91, 0.92, and 0.68 for physical, property, cultural, and economic threat, respectively).

#### Outcome Variables

##### Ethnic stereotypes

We used the same pool of items and instructions as in Study 1 for assessment of warmth and competence. In each group, both scales showed satisfactory reliability, with Cronbach’s alpha ranging from 0.72 to 0.90 (overall means of 0.85 and 0.86 for competence and warmth, respectively).

### Data Analysis

Data screening included checking for outliers and missing data. Also, we calculated a sum of attention check items to exclude cases if participants inattentively filled in the questionnaire. For the preliminary analysis, we used Cronbach’s alpha and Spearman-Brown coefficient for internal consistency, and correlation analysis.

To test the hypotheses, we used regression analysis. Finally, in the exploratory part of the study, we applied the networks analysis using the R package *qgraph* and EBICglasso procedure that provides only the most important empirical relationships in the data on the graphs excluding spurious relationship (Epskamp et al., 2012). Networks include graphical representations of the associations (edges) between variables (nodes). Using different edges thickness and colors, the networks show the regularized partial correlation between each pair of variables after accounting the shared variance in the network. Since not all nodes in a network are equally important in determining the network’s structure, we used three centrality measures: strength, closeness, and betweenness. A node is central (or important/influential) if (1) it has many strong direct connections (strength); (2) it is close (indirect connections) to all other nodes (closeness); and (3) it connects other nodes (betweenness) (Epskamp et al., 2012).

## Results

### Preliminary Analysis

The data had no missing values. The data had no observations that were consistent with the criterion for exclusion. The several outliers (from 2 to 6 cases, depending on the outcome variable), which were automatically excluded from the analyses, were detected by the Mahalanobis distance, Cook’s distance, and leverage value (Tabachnick and Fidell, 2018). The indicators of internal consistency showed satisfactory reliability, both Cronbach’s alpha and Spearman-Brown coefficient ranging from 0.58 to 0.97 (overall mean of 0.82).

**FIGURE 2:**
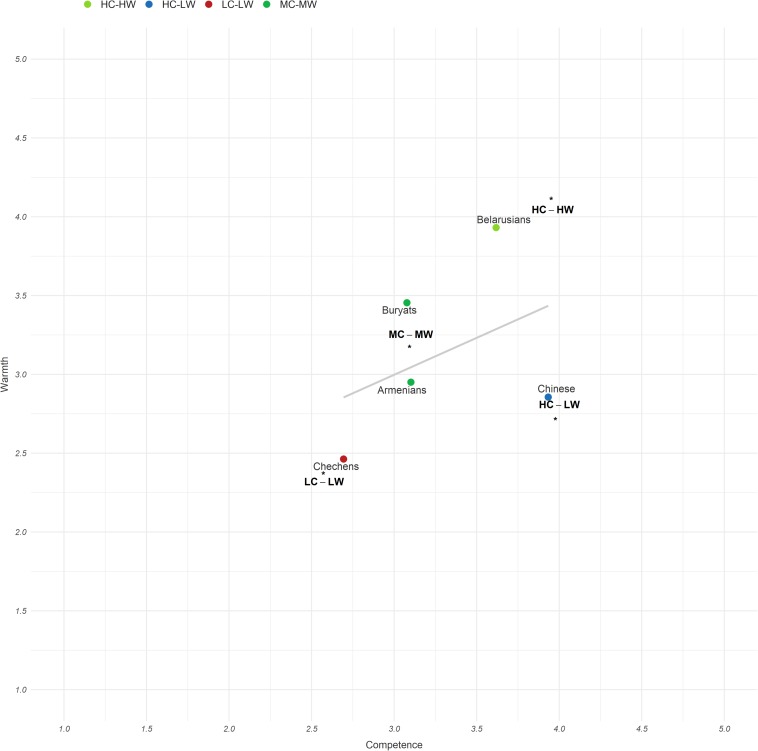
The distribution of the ethnic groups on the SCM map.

The combination of the cluster center means from Study 1 and groups means from Study 2 showed that the group positions in the space were fully replicated. The distribution of the ethnic groups on the SCM map appears in Figure 2. The paired sample *t*-test comparisons of competence and warmth means for each ethnic groups can be found in Table 2.

**TABLE 2:** Competence and Warmth means for each ethnic group, study 2.

	***M* (*SD*)**				
			
	**Competence**		**Warmth**	***t*(df)**	***d***
Belarusians (HC-HW)	3.62 (0.84)	<	3.93 (0.84)	−11.201(669)*	0.36
Armenians (MC-MW)	3.10 (0.95)	>	2.96 (0.98)	4.843(669)*	0.15
Buryats (LC-HW)	3.07 (0.92)	<	3.45 (0.94)	−14.285(669)*	0.41
Chechens (LC-LW)	2.70 (1.05)	>	2.46 (1.07)	8.315(669)*	0.22
Chinese (HC-LW)	3.93 (0.78)	>	2.86 (0.95)	29.687(669)*	1.23

^*^p < 0.001.

### Regression Analysis

The results of the regression analysis appear in Table 3 for competence and the Table 4 for warmth. The multicollinearity diagnostics showed an acceptable variance inflation factor for each predictor (VIF < 10), VIF ranging from 1.090 to 2.493 (overall mean of 1.563). The common explained variance of the stereotype content on the specific-group level ranged from 20 to 45%, overall means of 33 and 30% for competence and warmth, respectively. The common explained variance of the stereotype content on the cross-group level accounted for 43 and 41% for competence and warmth.

**TABLE 3:** Regression analysis predicting competence, study 2.

	**Belarusians (HC-HW)**		**Armenians (MC-MW)**		**Buryats (LC-HW)**		**Chechens (LC-LW)**		**Chinese (HC-LW)**		**Cross-group**	
						
	***r***	**β**	***r***	**β**	***r***	**β**	***r***	**β**	***r***	**β**	***r***	**β**
Status	0.54^∗∗∗^	0.49^∗∗∗^	0.51^∗∗∗^	0.45^∗∗∗^	0.56^∗∗∗^	0.53^∗∗∗^	0.56^∗∗∗^	0.46^∗∗∗^	0.43^∗∗∗^	0.40^∗∗∗^	0.62^∗∗∗^	0.58^∗∗∗^
Competition	−0.08^*^	–0.03	–0.15^∗∗∗^	–0.12^∗∗^	–0.04	0.02	–0.34^∗∗∗^	–0.17^∗∗∗^	–0.12^∗∗^	–0.12^∗∗^	–0.17^∗∗∗^	–0.09^∗∗^
Cultural distance	–0.12^∗∗^	–0.05	–0.22^∗∗∗^	–0.06	–0.22^∗∗∗^	–0.09^∗∗^	–0.26^∗∗∗^	–0.04	–0.02	0.01	–0.15^∗∗∗^	–0.02
Unfavorable region	–0.25^∗∗∗^	–0.05	–0.17^∗∗∗^	–0.01	–0.21^∗∗∗^	0.04	–0.30^∗∗∗^	–0.02	–0.18^∗∗∗^	–0.02	–0.25^∗∗∗^	–0.01
Physical threat	–0.18^∗∗∗^	0.04	–0.31^∗∗∗^	0.01	–0.21^∗∗∗^	–0.07	–0.42^∗∗∗^	–0.07	–0.17^∗∗∗^	–0.06	–0.30^∗∗∗^	–0.06
Property threat	–0.28^∗∗∗^	–0.16^∗∗∗^	–0.37^∗∗∗^	–0.22^∗∗∗^	–0.26^∗∗∗^	–0.12^∗∗^	–0.45^∗∗∗^	–0.13^∗∗^	–0.21^∗∗∗^	–0.07	–0.31^∗∗∗^	–0.11^∗∗^
Cultural threat	–0.16^∗∗∗^	–0.01	–0.24^∗∗∗^	–0.01	–0.12^∗∗^	0.02	–0.33^∗∗∗^	0.03	–0.21^∗∗∗^	–0.07	–0.23^∗∗∗^	–0.01
Economic threat	−0.10^∗^	–0.03	–0.13^∗∗∗^	0.04	–0.12^∗∗^	–0.04	–0.27^∗∗∗^	–0.05	−0−0.03	0.11^*^	–0.17^∗∗∗^	0.02
*R*^2^		0.33		0.34		0.35		0.42		0.23		0.43

^∗∗∗^*p* < 0.001; ^∗∗^*p* < 0.01; ^*^p < 0.05.

**TABLE 4:** Regression analysis predicting warmth, study 2.

	**Belarusians (HC-HW)**		**Armenians (MC-MW)**		**Buryats (LC-HW)**		**Chechens (LC-LW)**		**Chinese (HC-LW)**		**Cross-group**	
						
	***r***	**β**	***r***	**β**	***r***	**β**	***r***	**β**	***r***	**β**	***r***	**β**
Status	0.41^∗∗∗^	0.39^∗∗∗^	0.38^∗∗∗^	0.32^∗∗∗^	0.35^∗∗∗^	0.32^∗∗∗^	0.52^∗∗∗^	0.39^∗∗∗^	0.33^∗∗∗^	0.32^∗∗∗^	0.57^∗∗∗^	0.52^∗∗∗^
Competition	–0.22^∗∗∗^	–0.11^∗∗^	–0.27^∗∗∗^	–0.16^∗∗∗^	–0.20^∗∗∗^	−0.08^*^	–0.38^∗∗∗^	–0.14^∗∗∗^	–0.20^∗∗∗^	–0.17^∗∗∗^	–0.20^∗∗∗^	–0.09^∗∗^
Cultural distance	–0.29^∗∗∗^	–0.20^∗∗∗^	–0.27^∗∗∗^	–0.11^∗∗^	–0.26^∗∗∗^	–0.14^∗∗∗^	–0.33^∗∗∗^	–0.09^∗∗^	–0.15^∗∗∗^	–0.11^∗∗^	–0.25^∗∗∗^	–0.10^∗∗^
Unfavorable region	–0.14^∗∗∗^	0.04	–0.19^∗∗∗^	–0.03	–0.14^∗∗∗^	0.07	–0.27^∗∗∗^	0.04	−0.09^*^	0.06	–0.21^∗∗∗^	0.05
Physical threat	–0.25^∗∗∗^	0.01	–0.36^∗∗∗^	–0.03	–0.32^∗∗∗^	–0.16^∗∗^	–0.51^∗∗∗^	–0.17^∗∗∗^	–0.18^∗∗∗^	–0.02	–0.33^∗∗∗^	–0.06
Property threat	–0.29^∗∗∗^	–0.13^∗∗^	–0.39^∗∗∗^	–0.17^∗∗∗^	–0.32^∗∗∗^	−0.11^*^	–0.50^∗∗∗^	−0.11^*^	–0.26^∗∗∗^	–0.13^∗∗^	–0.35^∗∗∗^	–0.13^∗∗^
Cultural threat	–0.26^∗∗∗^	–0.05	–0.30^∗∗∗^	0.01	–0.23^∗∗∗^	–0.01	–0.40^∗∗∗^	–0.02	–0.22^∗∗∗^	–0.05	–0.28^∗∗∗^	–0.01
Economic threat	–0.16^∗∗∗^	–0.03	–0.27^∗∗∗^	–0.06	–0.21^∗∗∗^	–0.05	–0.35^∗∗∗^	–0.07	–0.12^∗∗^	0.06	–0.23^∗∗∗^	–0.02
*R*^2^		0.28		0.30		0.25		0.45		0.20		0.41

^∗∗∗^*p* < 0.001; ^∗∗^*p* < 0.01; ^*^p < 0.05.

#### Social Structure

Status positively predicted both competence and warmth across all the ethnic groups. Competition negatively predicted both competence and warmth across all the ethnic groups except the high-warmth groups (Belarusians and Buryats). The similar pattern of the relationships held for the cross-group level analysis. This result fits SCM predictions for status-competence and competition-warmth correlations, but it does not fit the specificity prediction that status-warmth and competition-competence correlations will be zero (H6a and H6b). However, the obtained result does fit the high warmth-competence correlation in these Russian data.

#### Cultural Distance and Structural Features of a Region

As in Study 1, cultural distance negatively predicted warmth on the specific- and cross-group levels. Also, cultural distance negatively predicted competence only for Buryats (LC-HW) (H7). Although the perceived structural features of a region correlated with competence and warmth across all the ethnic groups, this variance was shared with the other variables and did not add to prediction (H8).

#### Intergroup Threat

Predictions for intergroup threats were partially supported (H9a and H9b). Property threat negatively predicted both competence and warmth on the cross- and specific-group levels except Chinese (HC-LW). However, for Chinese, economic threat positively predicted competence; in the rest, economic threat did not add to prediction. As expected, physical threat negatively predicted warmth for the low-competence groups (Buryats and Chechens). Cultural threat did not add to prediction.

### Network Analysis

**FIGURE 3:**
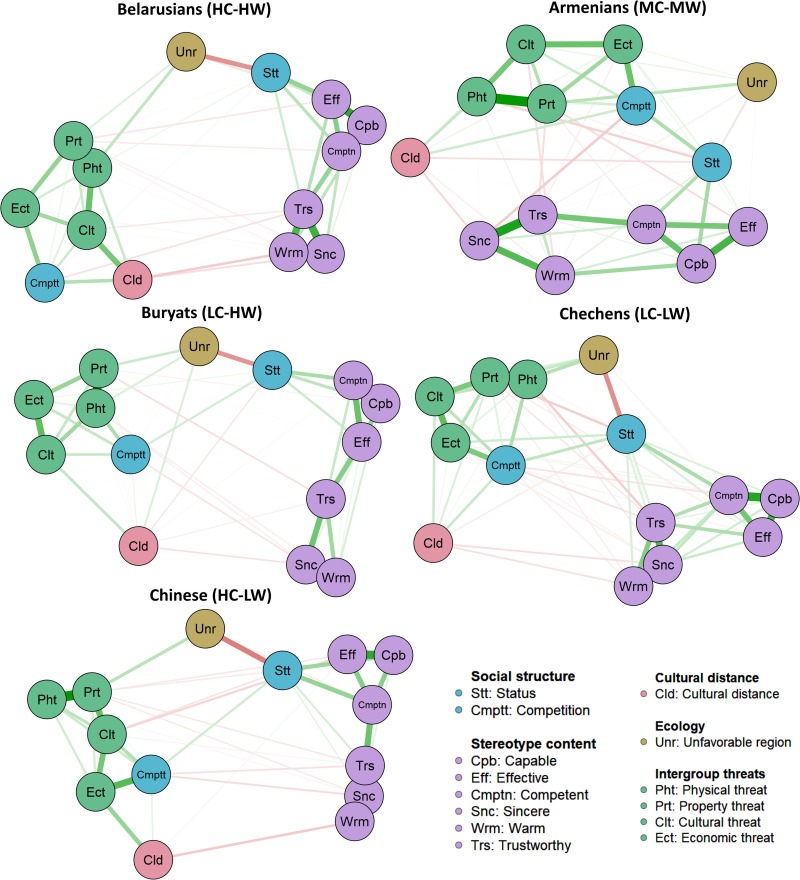
The ethnic stereotypes’ networks.

**FIGURE 4:**
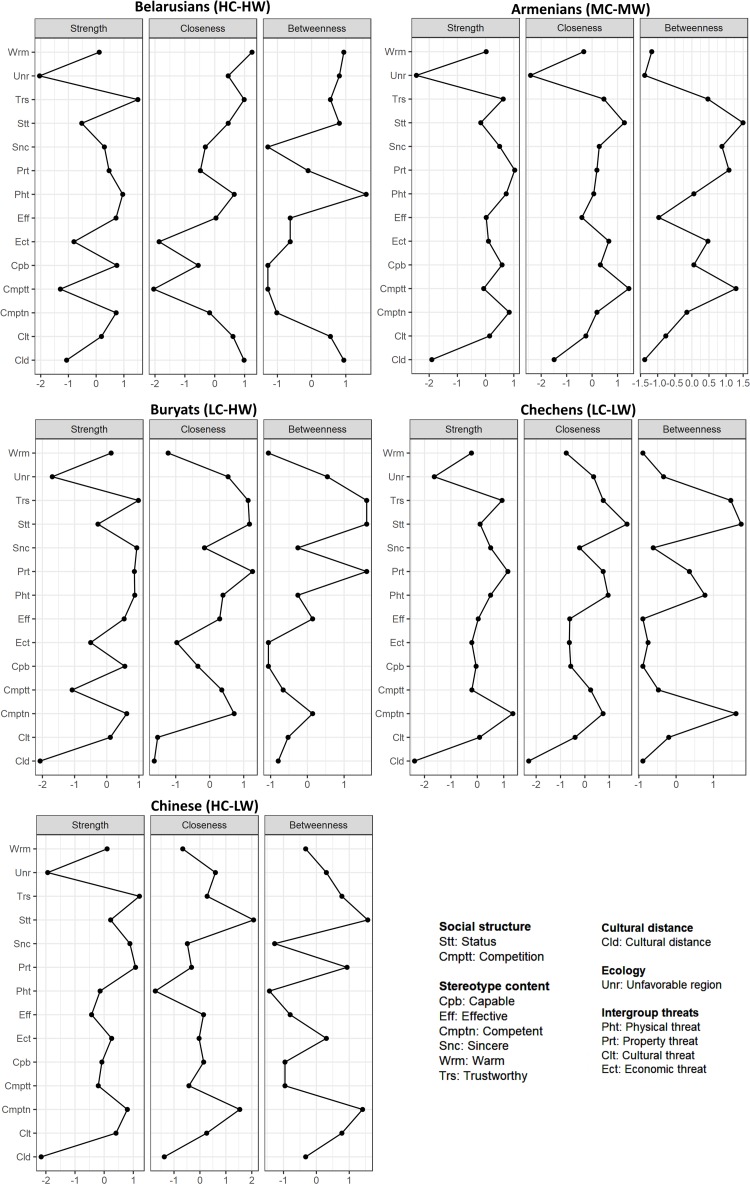
The standardized centrality measures of the ethnic stereotypes’ networks. For a correct interpretation, the values of the *X*-axis are standardized (*Z*-scores).

The networks and their centrality measures appear in Figures 3, 4, respectively (full-size images are available in the Supplementary Material S2). The centrality measures indicated that in the networks, principally status, competition, and property threat directly influence many other variables (or be influenced by them), communication over the clusters of nodes of networks is primarily through them, and they provide the fastest network changes. Trustworthy in the cluster of nodes of ethnic stereotypes had similar characteristics. The perceived structural features of a region and cultural distance also had connections in the networks but likely are more distal antecedents of the ethnic stereotypes. Also, in the network structures, different types of threats had strength of connections and closeness to cultural distance and competition depending on the specific group. In addition, the graphs showed sparsely connected networks, with competition close to the intergroup threats, while status was close to the ethnic stereotypes. In general, status connected the two communities: (a) threats including competition and (b) stereotype content.

## Discussion

This study tested several assumptions beyond the SCM, to compare some other approaches to ethnic stereotypes addressing the social structure, perceived cultural distance, perceived structural features of a region, and intergroup threats. The data supported our expectations to varying degrees.

### General Antecedents: Status, Competition, Cultural Distance, Region

Above all, one SCM antecedent, *status*, best predicted stereotype content. Furthermore, status was one of the central nodes of the ethnic stereotypes’ system networks. That indicated status is the robust proximal antecedent of the ethnic stereotypes in Russia. Status creates psychological distance and distinctions that are relatively automatic and spontaneous (Fiske et al., 2016). Status has an important role in the categorization of ethnic groups in Russia (Grigoryan, 2019; Grigoryan, under review). This can describe the strong hierarchy of Russian society and the high power distance that as a rule leads to high-status groups being perceived to have more positive traits (Minescu and Poppe, 2011). Moreover, the strong relationship between the status and competence can testify to a motivation for justifying the system (Oldmeadow and Fiske, 2007). The high correspondence between status and competence leads some models to treat them as a single stereotype dimension (Koch et al., 2016). Notably, however, the status-competence correlation is weaker in Russia and other post-communist states, though it still holds as a lay belief (Grigoryan et al., under review).

The other SCM antecedent, *competition*, also predicted warmth (but more weakly than status did) and likely included the variance of intergroup threats. The asymmetry in the status-competence and competition-warmth predictions was related to their position in stereotypes’ system networks. Status had the more proximal position to stereotype content than competition. Thus, the competition-to-stereotype-content link was, as a rule, confounded by the status or cultural distance associations. Moreover, competition also negatively predicted competence among groups with the moderate or low warmth. This protective or compensatory mechanism can appear in comparative settings (see Judd et al., 2005).

*Cultural distance* negatively predicted warmth. This corresponds to the assumption that culturally close groups are perceived as friendlier and evaluated more positively (Taylor, 1991; Muttarak, 2014; Alcott and Watt, 2017; see also Grigoryan, 2019). At the same time, the cultural distance negatively predicted competence only for Buryats (LC-HW), so compensation also appears.

The *structural features of a region* were not unique predictors at all. The direct association of unfavorable region with status, revealed in the network analysis, suggests that this predictor can be a socio-geographical marker of status, and this influences stereotypes confounded by the status.

### Group-Specific Antecedents

A consistent threat profile for some groups also emerged. Although *property threat* added to a unique component to the prediction of stereotype content and, unlike perceived cultural distance, rather together with status reflected some global estimate of evaluation. Property threat can be a marker for the groups tied to ethnic criminality; some ethnic groups in Russia are strictly associated with specific types of criminal activity (Arnold, 2016). So low status and property threat are likely to be a proxy to access intention, connecting the group with crime and lowering its evaluation. However, in general, the right to own property and the prohibition of theft have been widely regarded as an important component of morality that appears to be a cross-cultural universal (Curry et al., 2019).

*Physical threat* had a negative relationship with the warmth of low-competence groups. Ethnic groups perceived as incompetent were also perceived as more aggressive and conflictual (Phalet and Poppe, 1997).

*Economic threat* positively predicted competence for Chinese. Russian media often speak of China as the world’s leading economic competitor in the global market, and only skillful groups can represent a real threat of economic competition.

In general, the differences in the share of the explained variance and the importance of various predictors for the considered groups fit the differentiated threat approach (Cottrell and Neuberg, 2005; Meuleman et al., 2018; Landmann et al., 2019) and emphasizes the importance of macro-social factors—such as historical, political, geographical, and social—in explaining stereotypes.

### Groups in SCM Warmth-by-Competence Space

Finally, the target group positions in the space fully replicated Study 1 (also, the difference in means between competence and warmth for the groups was the same as in Study 1). Admittedly, the evaluations slightly varied, perhaps because using a different set of ethnic groups for comparison apparently evoked the compensation effect, such that the comparative context in which groups are perceived influences how they are judged (see Kervyn et al., 2008).

## General Discussion

In this research, we explained ethnic stereotypes in Russia based on the SCM framework. Russia is a heterogeneous region with its own specifics of interethnic relations. The interethnic relationships in Russia have their own long, unique history and contexts. At the same time, we managed to obtain results that show some universal patterns apart from cultural-specific ones.

First, the SCM warmth and competence ratings distinguished the groups from each other, though the two dimensions were moderately correlated. The research also showed well-established general mechanisms of religious and cultural similarity, ingroup/outgroup positions, and status. Perhaps the evolutionary source of the genesis of similarities (see e.g., Jones, 2018) and status relations (see e.g., Chapais, 2015) provide universal patterns across cultural contexts. To detect friend or foe and their potential agency could have a special adaptive (i.e., functional) purpose.

Apparently, competition to a greater extent includes two levels of threat: group-level threats (e.g., cultural and economic) while maintaining the potential of prediction for the individual level threats (e.g., physical and property). These findings with a focus on the differentiated threat approach can aid the further development of the theory of intergroup threats.

The importance of the context appeared again. The historical context (e.g., interethnic relations in the Soviet Union and after its collapse) and current geopolitical context (e.g., the Ukraine-Russia crisis, Russia-Turkey conflict) matter. So in conditions of unambivalent ethnic stereotypes, as in Russia, conflict situations likely cause contesting outgroup shifts to low evaluation on both competence and warmth dimensions. Our results with locations by using the official list of ethnic groups (*top-down approach*) mostly correspond to locations for spontaneously mentioned Russian social groups (*bottom-up approach*), except that those groups show all four quadrants and a lower warmth-competence correlation (see Grigoryan et al., under review).

### Limitations and Further Research

Independently of the distance between the cultures, cultures may themselves vary internally along a continuum of cultural homogeneity (e.g., Japan) – heterogeneity (e.g., Russia) (Ward and Geeraert, 2016). Moreover, in Study 2, we used the perceived cultural distance, which is related to perceivers’ own personality traits (emotionally more stable, more flexible individuals perceive less cultural distance), so reported distance is not a simple evaluation of objective cultural differences (Suanet and van de Vijver, 2009). Future studies could better conceptualize and operationalize the concept of cultural distance. Moreover, some differences in outgroup evaluations depend on the specific socio-cultural ingroups and outgroups (Ramsay and Pang, 2017; Meuleman et al., 2018). For example, some studies show that men have more ethnic stereotypes than women (Zick et al., 2008). Other aspects of intergroup similarity (discussed above) could provide additional details. Also promising is the idea of getting a complete SCM map in Russia combining ethnic and social groups, as in the United States (e.g., Lee and Fiske, 2006) (for Russia and several post-Soviet countries, see Grigoryan et al., under review, SI).

Also, in the Russian case, the respondent’s place of residence could matter. Russia has regions with predominantly ethnic Russian populations and so-called “national republics,” where the majority is represented by another ethnic group. In addition, different regions have a different number of certain immigrant groups. We assume that further cross-regional analysis could broaden understanding of some possible variation in the ethnic stereotypes in Russia. For example, Stanciu et al. (2017) provided some evidence about within-culture variation in the content of stereotypes.

This research did not fully reveal the potential of the differentiated threat approach. First of all, this was related to measurement issues since it is difficult to achieve an orthogonal measurement of threats using their wider set. In addition, the selection of other target groups (ethnic and social) could also help further develop the obtained results. For example, the fruitfulness of the differentiated threat approach to attitudes toward refugees was shown in the recent study in Germany (see Landmann et al., 2019). Moreover, this research did not fully reveal the potential of social ecology in explaining of stereotype content (in the future, Linssen and Hagendoorn (1994) could provide comparative perspective).

## Implications and Conclusion

The present work offers both theoretical and practical implications. On the first step of this research, we solved the task of forming the SCM map of ethnic stereotypes in Russia. Specific ethnic groups have stereotypes that vary more dramatically by culture (Fiske, 2017). Fiske and Durante (2016) noted that “planning interventions in difficult intergroup contexts can build on knowledge of how groups view each other. Coming into unfamiliar contexts can be eased by knowing the societal map of how groups locate in the SCM space. Just as geographic maps are helpful, so are cultural maps.” (p. 246). Thus, this research can be a starting point for future studies of interethnic relations in Russia from a comparative perspective. Attitudes toward immigrant and minority groups are likely to differ depending on the specific group, and thus studying broad attitudes toward immigrants or minority members in general will obfuscate some important details (Montreuil and Bourhis, 2001; Satherley and Sibley, 2016; Meuleman et al., 2018).

As noted, not only the complexity of intergroup relations but acculturation processes depend substantially on mutual interactions and expectations between members of dominant and non-dominant groups (e.g., van Oudenhoven et al., 1998; Arends-Tóth and van de Vijver, 2003; Berry, 2006; Brown and Zagefka, 2011; Horenczyk et al., 2013; Matera et al., 2015). Some acculturation models directly recognized this concept of mutuality (e.g., Bourhis et al., 1997; Piontkowski et al., 2002; Navas et al., 2005; Berry, 2006). Host societies have received less attention (Brown and Zagefka, 2011; Horenczyk et al., 2013), although often they provide the main tone in the process of mutual acculturation (Bourhis et al., 1997; Piontkowski et al., 2002), also in Russia (see Lebedeva et al., 2017). Acculturation expectations of host population can differ according to the origin of the immigrant group assessed and whether immigrant groups are valued or devalued; dominant group members “may be more likely to adopt acculturation orientations such as assimilationism and segregationism toward devalued immigrant outgroups against whom they already have negative stereotypes or whose culture and religion may be felt to differ considerably from their own” (Montreuil and Bourhis, 2001, p. 701). This can derive from the shared stereotype content among dominant group members, which may invoke their specific acculturation expectations (Lee and Fiske, 2006).

Stereotype content systematically relates to both acculturation perceptions and expectations (López-Rodríguez et al., 2014; López-Rodríguez and Zagefka, 2015; Alcott and Watt, 2017). The dominant group’s perceptions of immigrants’ acculturation strategies invoke the dominant group’s stereotypes about them (López-Rodríguez et al., 2014; Alcott and Watt, 2017); and at the same time, these stereotypes about immigrants invoke the dominant group’s acculturation preferences for them (López-Rodríguez et al., 2014; López-Rodríguez and Zagefka, 2015). The perception that non-dominant group members want to adopt the host culture led to more positive stereotype content about immigrants, regardless of whether or not immigrants maintain their original culture. Also in cultural distance, targets who did not adopt the host culture but maintained their own culture (i.e., those who chose separation) were perceived as the most culturally distant; distance, warmth, and competence are related. The host population’s desire for immigrants to adopt the host culture, become culturally similar, and integrate or assimilate, all these strategies may indicate their positive intent, which is the basis of the evaluation of warmth (Alcott and Watt, 2017). In Russia, for example, the concept of ethnic diaspora often carries a negative connotation, due to some association with the separation orientation.

Stereotype content about non-dominant group members invokes expectations about whether or not those others should maintain their original culture, but does necessarily invoke a desire for culture adoption (López-Rodríguez and Zagefka, 2015). The culture maintenance may be more diagnostic for assessing if someone represents a threat, so this dimension may be more sensitive to stereotypes especially about morality. In the SCM, (1) morality can be a force leading to cohesiveness within groups and the exclusion of outgroups (presumed to have different interests than the ingroups); this might relate to group survival, as a protective mechanism against intergroup threats; (2) morality facilitates the coordination among members group in order to maximize benefits for individual members and the whole group. So the SCM map allows selecting targeted groups depending on their position in the SCM space, which can suggest the various acculturation expectations of the host population (e.g., integration vs. assimilation or segregation) in different domains (e.g., public domains: work, contact, etc., and private domains: family, values, etc., see Grigoryev and van de Vijver, 2018). The approach of combining group- and domain-specificity of acculturation will enrich knowledge of how to improve the mutual adjustment in plural societies. Also, for the acculturation area in general, the findings suggest not to neglect the social structure when considering interethnic relations.

In addition, the implications for the SCM include the importance of status-competence, as well as a role for beliefs (religious and cultural), both featured in the ABC model^12^ (see Koch et al., 2016). The SCM also needs to continue including both tangible and intangible threats as competition, consistent with the Intergroup Threat Theory (Stephan and Mealy, 2011). Nevertheless, many widely shared SCM principles appear in Russia’s distinctive context.

## Data Availability

The datasets generated for this study are available on request to the corresponding author.

## Author Contributions

DG initiated the project, coordinated all the stages, developed the design of the manuscript and instruments, and involved in the data analysis, reporting, and drafting of the manuscript. SF was involved in the conceptualization of the study, revised it critically, and was involved in drafting of the manuscript. AB was involved in the conceptualization of the study, choice, development of the instruments, and drafting of the manuscript.

## Conflict of Interest Statement

The authors declare that the research was conducted in the absence of any commercial or financial relationships that could be construed as a potential conflict of interest.

## Footnotes

1 High competence and high warmth (HC-HW), high competence and low warmth (HC-LW), low competence and high warmth (LC-HW), and low competence and low warmth (LC-LW).

2 Admiration (admire and proud) for HC-HW, envy (envious and jealous) for HC-LW, pity (pity and sympathy) for LC-HW, and contempt (contempt and disgust) for LC-LW. All of that, according to the behavior from intergroup affect and stereotypes perspective, predict distinct behaviors: active facilitation (e.g., help and protect) for HC-HW and LC-HW, passive facilitation (e.g., cooperate with and associate with) for HC-HW and HC-LW, active harm (e.g., fight and attack) for HC-LW and LC-LW, and passive harm (e.g., exclude and demean) for LC-HW and LC-LW. Earliest works on stereotypes also suggested that they provide social norms for prejudice and discrimination and can serve as justification for reactions toward outgroups (e.g., Stangor and Schaller, 1996; Jost and Hamilton, 2005).

3 The second largest group is the Tatars (3.9%), next, Ukrainians, Bashkirs, Chuvashs, and Chechens (about 1% of the population for each group). The remaining ethnic groups represent less than 1% of the population for each group. See Supplementary Table S1 in the Supplementary Materials.

4 Gini coefficients were retrieved from the Central Intelligence Agency-The World Factbook, https://www.cia.gov/library/publications/the-world-factbook/fields/2172.html and GPI scores from the Institute for Economics and Peace, http://economicsandpeace.org/reports/ (July 15, 2018).

5 These differences in power distance, individualism, masculinity, uncertainty avoidance, long-term orientation, and indulgence can be tracked on the Hofstede Insights’ website, https://www.hofstede-insights.com/country-comparison/russia,the-usa/ (July 15, 2018).

6 Verbatim: *“Off the top of your head, what various types of people do you think today’s society categorizes into groups (i.e., based on ability, age, ethnicity, gender, occupation, race, religion, etc.)? Please list 8 to 16 groups.”* See Fiske et al., 2002, for more detail.

7 This procedure was in line with Russian regulations; as per university and national Russian regulations, no ethics clearance was required for this type of survey research (if it did not include medical data).

8 This information is available in the Supplementary Material S1.

9 Moscow and the Moscow region, unlike other regions of the Central Federal District of Russia, are more ethnically heterogeneous and the main recipient of migrants, while the population of selected regions is about 95% ethnic Russians according to census data.

10 The informed consent of the participants was also implied through survey completion. This procedure was in line with Russian regulations; as per university and national Russian regulations, no ethics clearance was required for this type of survey research (if it did not include medical data).

11 To estimate the reliability of measures consisting of two items, the Spearman-Brown coefficient is used.

12 The ABC (Agency-Beliefs-Communion) model used a more data-driven approach and incorporated the concept of status into its agency component (and excluded ability from it). This status + assertiveness criterion for agency dimension and a novel beliefs dimension came from the principal components that emerged from ratings of social groups in a variety of traits. A comparison of the methods and assumptions of the SCM and ABC models suggests that the SCM likely uses a more interpersonal/psychological approach, while the ABC takes a more abstract/sociological perspective. For example, the SCM is based on functional theories about interpersonal interaction and is based solely on the direct rating of a few groups while the ABC asks participants to think about many different groups from the abstract perspective of “intergroup similarities”, which might evoke a more sociological organization of social groups (Fiske et al., 2019).

## References

[B1] Alcott Y. D. Watt S. E. (2017). Acculturation strategy and racial group in the perception of immigrants. *J. Pacific Rim Psychol.* 11:e1 10.1017/prp.2017.2

[B2] Allik J. Mõttus R. Realo A. (2010). Does national character reflect mean personality traits when both are measured by the same instrument? *J. Res. Pers.* 44 62–69. 10.1016/j.jrp.2009.10.008

[B3] Allport G. W. (1954). *The Nature of Prejudice.* Cambridge: Addison-Wesley.

[B4] Arends-Tóth J. van de Vijver F. (2003). Multiculturalism and acculturation: views of dutch and turkish-dutch. *Eur. J. Soc. Psychol.* 33 249–266. 10.1002/ejsp.143

[B5] Arnold R. (2016). *Russian Nationalism and Ethnic Violence: Symbolic Violence, Lynching, Pogrom and Massacre.* New York, NY: Routledge.

[B6] Asbrock F. (2010). Stereotypes of social groups in Germany in terms of warmth and competence. *Soc. Psychol.* 41 76–81. 10.1027/1864-9335/a000011

[B7] Balzer M. M. (ed.) (2015). *Religion and Politics in Russia.* New York, NY: Routledge.

[B8] Bergsieker H. B. Leslie L. M. Constantine V. S. Fiske S. T. (2012). Stereotyping by omission: eliminate the negative, accentuate the positive. *J. Pers. Soc. Psychol.* 102 1214–1238. 10.1037/a0027717 22448889 PMC4251455

[B9] Berry J. W. (2006). Mutual attitudes among immigrants and ethnocultural groups in canada. *Int. J. Intercult. Relat.* 30 719–734. 10.1016/j.ijintrel.2006.06.004 30686038

[B10] Bessudnov A. (2016). Ethnic hierarchy and public attitudes towards immigrants in Russia. *Eur. Sociol. Rev.* 32 567–580. 10.1093/esr/jcw002

[B11] Binggeli S. Krings F. Sczesny S. (2014). Stereotype content associated with immigrant groups in Switzerland. *Swiss J. Psychol.* 73 123–133. 10.1024/1421-0185/a000133

[B12] Bourhis R. Y. Moïse L. C. Perreault S. Senecal S. (1997). Towards an interactive acculturation model: a social psychological approach. *Int. J. Psychol.* 32 369–386. 10.1080/002075997400629

[B13] Brown R. Zagefka H. (2011). “The dynamics of acculturation: an intergroup perspective,” in *Advances in Experimental Social Psychology.* eds Olson J. M. Zanna M. P. (Cambridge, MA: Academic Press), 10.1016/B978-0-12-385522-0.00003-2

[B14] Burkley E. Durante F. Fiske S. T. Burkley M. Andrade A. (2017). Structure and content of native american stereotypic subgroups: not just (ig)noble. *Cult. Divers. Ethnic Minor. Psychol.* 23 209–219. 10.1037/cdp0000100 27429064

[B15] Bye H. H. Herrebrøden H. Hjetland G. J. Røyset G. Ø Westby L. L. (2014). Stereotypes of norwegian social groups. *Scand. J. Psychol.* 55 469–476. 10.1111/sjop.12141 24975918 PMC4282792

[B16] Chapais B. (2015). Competence and the evolutionary origins of status and power in humans. *Hum. Nat.* 26 161–183. 10.1007/s12110-015-9227-6 25947621

[B17] Costa-Lopes R. Vala J. Judd C. (2012). Similarity and dissimilarity in intergroup relations: different dimensions, different processes. *Revue int. de psychol. Soc.* 25 31–65.

[B18] Cottrell C. A. Neuberg S. L. (2005). Different emotional reactions to different groups: a sociofunctional threat-based approach to “prejudice”. *J. Pers. Soc. Psychol.* 88 770–789. 10.1037/0022-3514.88.5.770 15898874

[B19] Cuddy A. J. C. Fiske S. T. Glick P. (2008). Warmth and competence as universal dimensions of social perception: the stereotype content model and the BIAS map. *Adv. Exp. Soc. Psychol.* 40 61–149. 10.1016/S0065-2601(07)00002-0

[B20] Cuddy A. J. C. Fiske S. T. Kwan V. S. Y. Glick P. Demoulin S. Leyens J.-P. (2009). Stereotype content model across cultures: towards universal similarities and some differences. *Br. J. Soc. Psychol.* 48 1–33. 10.1348/014466608X314935 19178758 PMC3912751

[B21] Curry O. S. Mullins D. A. Whitehouse H. (2019). Is it good to cooperate? testing the theory of morality-as-cooperation in 60 societies. *Curr. Anthropol.* 60 47–69. 10.1086/701478

[B22] De Schryver M. Vindevogel S. Rasmussen A. E. Cramer A. O. J. (2015). Unpacking constructs: a network approach for studying war exposure, daily stressors and post-traumatic stress disorder. *Front. Psychol.* 6:1896. 10.3389/fpsyg.2015.01896 26733901 PMC4679872

[B23] Durante F. Fiske S. T. Gelfand M. J. Crippa F. Suttora C. Stillwell A. (2017). Ambivalent stereotypes link to peace, conflict, and inequality across 38 nations. *Proc. Natl. Acad. Sci. U.S.A.* 114 669–674. 10.1073/pnas.1611874114 28069955 PMC5278477

[B24] Durante F. Fiske S. T. Kervyn N. Cuddy A. J. C. Akande A. D. Adetoun B. E. (2013). Nations’ income inequality predicts ambivalence in stereotype content: how societies mind the gap. *Br. J. Soc. Psychol.* 52 726–746. 10.1111/bjso.12005 23039178 PMC3855559

[B25] Epskamp S. Cramer A. O. J. Waldorp L. J. Schmittmann V. D. Borsboom D. (2012). Qgraph: network visualization of relationships in psychometric data. *J. Stat. Softw.* 48 1–18. 10.18637/jss.v048.i04

[B26] Fiske S. T. (2015). Intergroup biases: a focus on stereotype content. *Curr. Opin. Behav. Sci.* 3 45–50. 10.1016/j.cobeha.2015.01.010 27453920 PMC4955357

[B27] Fiske S. T. (2017). Prejudices in cultural contexts: shared stereotypes (gender, age) versus variable stereotypes (race, ethnicity, religion). *Perspect. Psychol. Sci.* 12 791–799. 10.1177/1745691617708204 28972839 PMC5657003

[B28] Fiske S. T. (2018). Stereotype content: warmth and competence endure. *Curr. Dir. Psychol. Sci.* 27 67–73. 10.1177/0963721417738825 29755213 PMC5945217

[B29] Fiske S. T. Cuddy A. J. C. Glick P. (2007). Universal dimensions of social cognition: warmth and competence. *Trends Cogn. Sci.* 11 77–83. 10.1016/j.tics.2006.11.005 17188552

[B30] Fiske S. T. Cuddy A. J. C. Glick P. Xu J. (2002). A model of (often mixed) stereotype content: competence and warmth respectively follow from perceived status and competition. *J. Personal. Soc. Psychol.* 82 878–902. 10.1037/0022-3514.82.6.878 12051578

[B31] Fiske S. T. Dupree C. H. Nicolas G. Swencionis J. K. (2016). Status, power, and intergroup relations: the personal is the societal. *Curr. Opin. Psychol.* 11 44–48. 10.1016/j.copsyc.2016.05.012 27453923 PMC4955850

[B32] Fiske S. T. Durante F. (2016). “Stereotype content across cultures: Variations on a few themes,” in *Handbook of Advances in Culture and Psychology*, eds Gelfand M. J. Chiu C.-Y. Hong Y.-Y. (New York, NY: Oxford University Press), 209–258. 10.1093/acprof:oso/9780190458850.003.0005

[B33] Fiske S. T. Nicolas G. Bai X. (2019). “Stereotype content model: How we make sense of individuals and groups,” in *Social Psychology: Handbook of Basic Principles*, 2nd Edn, eds van Lange P. A. M. Higgins E. T. Kruglanski A. W. (New York, NY: Guilford).

[B34] Galchenko I. van de Vijver F. (2007). The role of perceived cultural distance in the acculturation of exchange students in Russia. *Int. J. Int. Relations* 31 181–197. 10.1016/j.ijintrel.2006.03.004

[B35] Grigoryan L. K. (2019). Perceived similarity in multiple categorization. *Appl. Psychol.* (in press). 10.1111/apps.12202

[B36] Grigoryev D. Batkhina A. van de Vijver F. Berry J. W. (2019). Towards an integration of models of discrimination of immigrants: from ultimate (functional) to proximate (sociofunctional) explanations. *J. Int. Migr. Integr.* (in press). 10.1007/s12134-019-00677-w

[B37] Grigoryev D. van de Vijver F. (2018). Acculturation expectation profiles of Russian majority group members and their intergroup attitudes. *Int. J. Intercult. Relat.* 64 90–99. 10.1016/j.ijintrel.2018.03.001

[B38] Grigoryev D. van de Vijver F. Batkhina A. (2018). Discordance of acculturation attitudes of the host population and their dealing with immigrants. *J. Intercult. Commun. Res.* 47 491–509. 10.1080/17475759.2018.1497678

[B39] Hagendoorn L. (1995). Intergroup biases in multiple group systems: the perception of ethnic hierarchies. *Eur. Rev. Soc. Psychol.* 6 199–228. 10.1080/14792779443000058

[B40] Hagendoorn L. Drogendijk R. Tumanov S. Hraba J. (1998). Inter-ethnic preferences and ethnic hierarchies in the former Soviet Union. *Int. J. Intercult. Relat.* 22 483–503. 10.1016/S0147-1767(98)00020-0

[B41] Hewstone M. Rubin M. Willis H. (2002). Intergroup bias. *Annu. Rev. Psychol.* 53 575–604. 10.1146/annurev.psych.53.100901.135109 11752497

[B42] Hofstede G. Hofstede G. J. Minkov M. (2010). *Cultures and Organizations: Software of the Mind. Intercultural Cooperation and Its Importance for Survival*, 3 Edn New York, NY: McGraw-Hill.

[B43] Horenczyk G. Jasinskaja-Lahti I. Sam D. L. Vedder P. (2013). Mutuality in acculturation: toward an integration. *Z. Psychol.* 221 205–213. 10.1027/2151-2604/a000150

[B44] Inglehart R. F. (2018). “Modernization, existential security, and cultural change: Reshaping human motivations and society,” in *Handbook of Advances in Culture and Psychology*, eds Gelfand M. J. Chiu C.-Y. Hong Y.-Y. (Oxford: Oxford University Press).

[B45] Janssens H. Verkuyten M. Khan A. (2015). Perceived social structural relations and group stereotypes: a test of the stereotype content model in Malaysia. *Asian J. Soc. Psychol.* 18 52–61. 10.1111/ajsp.12077

[B46] Jones D. (2018). Kin selection and ethnic group selection. *Evol. Hum. Behav.* 39 9–18. 10.1016/j.evolhumbehav.2017.08.004

[B47] Jost J. T. Hamilton D. L. (2005). “Stereotypes in our culture,” in *On the Nature of Prejudice:Fifty Years after Allport.* eds Dovidio J. F. Glick P. Rudman L. A. (Oxford: Blackwell).

[B48] Judd C. M. James-Hawkins L. Yzerbyt V. Y. Kashima Y. (2005). Fundamental dimensions of social judgment: understanding the relations between judgments of competence and warmth. *J. Pers. Soc. Psychol.* 89 899–913. 10.1037/0022-3514.89.6.899 16393023

[B49] Kervyn N. Fiske S. T. Yzerbyt V. (2015). Forecasting the primary dimension of social perception: symbolic and realistic threats together predict warmth in the stereotype content model. *Soc. Psychol.* 46 36–45. 10.1027/1864-9335/a000219 30555596 PMC6294451

[B50] Kervyn N. Fiske S. T. Yzerbyt V. Y. (2013). Integrating the stereotype content model (warmth and competence) and the Osgood semantic differential (evaluation, potency, and activity). *Eur. J. Soc. Psychol.* 43 673–681. 10.1002/ejsp.1978 26120217 PMC4479118

[B51] Kervyn N. Yzerbyt V. Y. Demoulin S. Judd C. M. (2008). Competence and warmth in context: the compensatory nature of stereotypic views of national groups. *Eur. J. Soc. Psychol.* 38 1175–1183. 10.1002/ejsp.526

[B52] Khaptsova A. Klyass M. Chuprikov B. (2018). Three hypotheses of multiculturalism in three days: depiction of 46 ethnic minorities in Russian online-media. *Psychol. J. Higher School Econ.* 15 346–367. 10.17323/1813-8918-2018-2-346-367

[B53] Koch A. Imhoff R. Dotsch R. Unkelbach C. Alves H. (2016). The ABC of stereotypes about groups: agency/socio-economic success, conservative-progressive beliefs, and communion. *J. Pers. Soc. Psychol.* 110 675–709. 10.1037/pspa0000046 27176773

[B54] Koenig A. M. Eagly A. H. (2019). Typical roles and intergroup relations shape stereotypes: how understanding social structure clarifies the origins of stereotype content. *Soc. Psychol. Q.* 82 205–230. 10.1177/0190272519850766 10.1177/0190272519850766

[B55] Kotzur P. F. Friehs M.-T. Asbrock F. van Zalk M. H. W. (2019). Stereotype content of refugee subgroups in Germany. *Eur. J. Soc. Psychol.* (in press). 10.1002/ejsp.2585

[B56] Kotzur P. F. Schäfer S. J. Wagner U. (2018). Meeting a nice asylum seeker: intergroup contact changes stereotype content perceptions and associated emotional prejudices, and encourages solidarity-based collective action intentions. *Br. J. Soc. Psychol.* [Epub ahead of print]. 10.1111/bjso.12304 30512181

[B57] Landmann H. Gaschler R. Rohmann A. (2019). What is threatening about refugees? identifying different types of threat and their association with emotional responses and attitudes towards refugee migration. *Eur. J. Soc. Psychol.* (in press). 10.1002/ejsp.2593

[B58] Lebedeva N. Galyapina V. Lepshokova Z. Ryabichenko T. (2017). “Intercultural relations in Russia,” in *Mutual Intercultural Relations*, ed. Berry J. W. (Cambridge: Cambridge University Press), 10.1017/9781316875032.002

[B59] Lee T. L. Fiske S. T. (2006). Not an outgroup, not yet an ingroup: immigrants in the stereotype content model. *Int. J. Intercult. Relat.* 30 751–768. 10.1016/j.ijintrel.2006.06.005

[B60] Linssen H. Hagendoorn L. (1994). Social and geographical factors in the explanation of the content of european nationality stereotypes. *Br. J. Soc. Psychol.* 33 165–182. 10.1111/j.2044-8309.1994.tb01016.x

[B61] López-Rodríguez L. Zagefka H. (2015). The effects of stereotype content on acculturation preferences and prosocial tendencies: the prominent role of morality. *Int. J. Intercult. Relat.* 45 36–46. 10.1016/j.ijintrel.2014.12.006

[B62] López-Rodríguez L. Zagefka H. Navas M. Cuadrado I. (2014). Explaining majority members’ acculturation preferences for minority members: a mediation model. *Int. J. Intercult. Relat.* 38 36–46. 10.1016/j.ijintrel.2013.07.001

[B63] Matera C. Stefanile C. Brown R. (2015). Majority–minority acculturation preferences concordance as an antecedent of attitudes towards immigrants: the mediating role of perceived symbolic threat and metastereotypes. *Int. J. Intercult. Relat.* 45 96–103. 10.1016/j.ijintrel.2015.02.001

[B64] McCrae R. R. Terracciano A. Realo A. Allik J. (2007). Climatic warmth and national wealth: some culture-level determinants of national character stereotypes. *Eur. J. Pers.* 21 953–976. 10.1002/per.647 20046546 PMC2800797

[B65] Meuleman B. Abts K. Slootmaeckers K. Meeusen C. (2018). Differentiated threat and the genesis of prejudice: group-specific antecedents of homonegativity, islamophobia, anti-semitism, and anti-immigrant attitudes. *Soc. Probl.* 66 222–244. 10.1093/socpro/spy002

[B66] Minescu A. Hagendoorn L. Poppe E. (2008). Types of identification and intergroup differentiation in the russian federation. *J. Soc. Issues* 64, 321–342. 10.1111/j.1540-4560.2008.00564.x

[B67] Minescu A. Poppe E. (2011). Intergroup conflict in Russia: testing the group position model. *Soc. Psychol. Q.* 74 166–191. 10.1177/0190272511408057

[B68] Montreuil A. Bourhis R. Y. (2001). Majority acculturation orientations toward “valued” and “devalued” immigrants. *J. Cross Cult. Psychol.* 32 698–719. 10.1177/0022022101032006004

[B69] Muttarak R. (2014). Generation, ethnic and religious diversity in friendship choice: exploring interethnic close ties in Britain. *Ethn. Racial Stud.* 37 71–98. 10.1080/01419870.2014.844844

[B70] Navas M. García M. C. Sánchez J. Rojas A. J. Pumares P. Fernández J. S. (2005). Relative acculturation extended model (RAEM): new contributions with regard to the study of acculturation. *Int. J. Intercult. Relat.* 29 21–37. 10.1016/j.ijintrel.2005.04.001

[B71] Oishi S. Graham J. (2010). Social ecology: lost and found in psychological science. *Perspect. Psychol. Sci.* 5 356–377. 10.1177/1745691610374588 26162183

[B72] Oldmeadow J. Fiske S. T. (2007). System-justifying ideologies moderate status = competence stereotypes: roles for belief in a just world and social dominance orientation. *Eur. J. Soc. Psychol.* 37 1135–1148. 10.1002/ejsp.428

[B73] Phalet K. Poppe E. (1997). Competence and morality dimensions of national and ethnic stereotypes: a study in six eastern-european countries. *Eur. J. Soc. Psychol.* 27 703–723. 10.1002/(SICI)1099-0992(199711/12)27:6<703::AID-EJSP841<3.0.CO;2-K

[B74] Piontkowski U. Rohmann A. Florack A. (2002). Concordance of acculturation attitudes and perceived threat. *Group Process. Intergroup Relat.* 5 221–232. 10.1177/1368430202005003003 18272803

[B75] Poppe E. (2001). Effects of changes in GNP and perceived group characteristics on national and ethnic stereotypes in Central and Eastern Europe. *J. Appl. Soc. Psychol.* 31 1689–1708. 10.1111/j.1559-1816.2001.tb02746.x

[B76] Ramsay J. E. Pang J. S. (2017). Anti-immigrant prejudice in rising East Asia: a stereotype content and integrated threat analysis. *Polit. Psychol.* 38 227–244. 10.1111/pops.12312

[B77] Safronov S. (2015). Russian population ethnic structure: trends and transformations. *Balt. Region* 3 106–120. 10.5922/2079-8555-2015-3-9

[B78] Satherley N. Sibley C. G. (2016). A Dual process model of attitudes toward immigration: predicting intergroup and international relations with China. *Int. J. Intercult. Relat.* 53 72–82. 10.1016/j.ijintrel.2016.05.008

[B79] Sayans-Jiménez P. Cuadrado I. Rojas A. J. Barrada J. R. (2017). Extracting the evaluations of stereotypes: bi-factor model of the stereotype content structure. *Front. Psychol* 8:1692. 10.3389/fpsyg.2017.01692 29085313 PMC5649216

[B80] Sibley C. G. Stewart K. Houkamau C. Manuela S. Perry R. Wootton L. W. (2011). Ethnic group stereotypes in New Zealand. *NZ. J. Psychol.* 40 25–36.

[B81] Stanciu A. Cohrs J. C. Hanke K. Gavreliuc A. (2017). Within-culture variation in the content of stereotypes: application and development of the stereotype content model in an Eastern European culture. *J. Soc. Psychol.* 157 611–628. 10.1080/00224545.2016.1262812 27874317

[B82] Stangor C. Schaller M. (1996). “Stereotypes as individual and collective representations,” in *Stereotypes and Stereotyping*, eds Macrae C. N. Stangor C. Hewstone M. (New York, NY: Guilford Press), 3–40.

[B83] Stephan W. G. Mealy M. D. (2011). “Intergroup Threat Theory,” in *The Encyclopedia of Peace Psychology*, ed. Christie D. J. (Hoboken, NJ: Wiley-Blackwell), 10.1002/9780470672532.wbepp139

[B84] Stephan W. G. Stephan C. W. (2000). “An integrated threat theory of prejudice,” in *Reducing Prejudice and Discrimination*, ed. Oskamp S. (Hillsdale, NJ: Erlbaum), 23–46.

[B85] Suanet I. van de Vijver F. (2009). Perceived cultural distance and acculturation among exchange students in Russia. *J. Commun. Appl. Soc. Psychol.* 19 182–197. 10.1002/casp.989

[B86] Tabachnick B. G. Fidell L. S. (2018). *Using Multivariate Statistics*, 7th Edn London: Pearson.

[B87] Taylor D. M. (1991). “The social psychology of racial and cultural diversity: Issues of assimilation and multiculturalism,” in *Bilingualism, Multiculturalism and Second-Language Learning*, ed. Reynolds A. G. (Hillsdale, NJ: Lawrence Erlbaum), 1–19.

[B88] Tishkov V. (1994). *Nationalities and Conflicting Ethnicity in Post-Communist Russia.* Geneva: United Nations Research Institute For Social Development.

[B89] Vaes J. Paladino M. P. (2010). The uniquely human content of stereotypes. *Group Process. Intergroup Relat.* 13 23–39. 10.1177/1368430209347331

[B90] van Osch Y. M. J. Breugelmans S. M. (2012). Perceived intergroup difference as an organizing principle of intercultural attitudes and acculturation attitudes. *J. Cross Cult. Psychol.* 43 801–821. 10.1177/0022022111407688

[B91] van Oudenhoven J. P. Prins K. S. Buunk B. P. (1998). Attitudes of minority and majority members towards adaptation of immigrants. *Eur. J. Soc. Psychol.* 28 995–1013. 10.1002/(SICI)1099-0992(1998110)28:6<995::AID-EJSP908<3.0.CO;2-8

[B92] Ward C. Geeraert N. (2016). Advancing acculturation theory and research: the acculturation process in its ecological context. *Curr. Opin. Psychol.* 8 98–104. 10.1016/j.copsyc.2015.09.021 29506811

[B93] Ward C. Szabo A. Stuart J. (2017). “Prejudice against immigrants in multicultural societies,” in *The Cambridge Handbook of the Psychology of Prejudice*, eds Sibley C. G. Barlow F. K. (Cambridge: Cambridge University Press), 10.1017/9781316161579.018

[B94] Williams K. E. G. Sng O. Neuberg S. L. (2016). Ecology-driven stereotypes override race stereotypes. *Proc. Natl. Acad. Sci. U.S.A.* 113 310–315. 10.1073/pnas.1519401113 26712013 PMC4720338

[B95] Willis G. B. (2004). *Cognitive Interviewing: A Tool for Improving Questionnaire Design.* Thousand Oaks, CA: Sage Publications.

[B96] Zick A. Wolf C. Kupper B. Davidov E. Schmidt P. (2008). The syndrome of group-focused enmity: the interrelation of prejudices tested with multiple cross-sectional and panel data. *J. Soc. Issues* 64 363–383. 10.1111/j.1540-4560.2008.00566.x

