# Supplementary material for: Mapping Ethnic Stereotypes and Their Antecedents in Russia: The Stereotype Content Model
Source: Front Psychol. 2019 Jul 16;10:1643. doi: 10.3389/fpsyg.2019.01643 (PMC6646730; doi:10.3389/fpsyg.2019.01643)
Supplement: Supplementary file 1 [file Table_1.DOC]

*Table S1. Ethnic Groups according to Census Data for 2010*

| Group | Language family | Main religion | Main area | Number | % |
| --- | --- | --- | --- | --- | --- |
| Russians | Indo-European | Eastern Orthodox Christianity | European Russia | 111,016,896 | 80.9% |
| Tatars | Turkic | Sunni Islam | European Russia | 5,310,649 | 3.9% |
| Ukrainians | Indo-European | Eastern Orthodox Christianity | European Russia | 1,927,888 | 1.4% |
| Bashkirs | Turkic | Sunni Islam | European Russia | 1,584,554 | 1.15% |
| Chuvashs | Turkic | Eastern Orthodox Christianity | European Russia | 1,435,872 | 1.05% |
| **Chechens** | **Northeast Caucasian** | **Sunni Islam** | **Caucasus** | **1,431,360** | **1.04%** |
| **Armenians** | **Indo-European** | **Oriental Orthodox Christianity** | **Caucasus** | **1,182,388** | **0.86%** |
| Avars | Northeast Caucasian | Sunni Islam | Caucasus | 912,090 | 0.66% |
| Mordvins | Uralic | Eastern Orthodox Christianity | European Russia | 744,237 | 0.54% |
| Kazakhs | Turkic | Sunni Islam | Central Asia | 647,732 | 0.47% |
| Azerbaijanis | Turkic | Shia Islam | Caucasus | 603,070 | 0.44% |
| Dargins | Northeast Caucasian | Sunni Islam | Caucasus | 589,386 | 0.43% |
| Udmurts | Uralic | Eastern Orthodox Christianity | European Russia | 552,299 | 0.40% |
| Ossetians | Indo-European | Eastern Orthodox Christianity | Caucasus | 528,515 | 0.38% |
| **Belarusians** | **Indo-European** | **Eastern Orthodox Christianity** | **European Russia** | **521,443** | **0.38%** |
| Kabardians | Northwest Caucasian | Sunni Islam | Caucasus | 516,826 | 0.38% |
| Yakuts | Turkic | Eastern Orthodox Christianity | Siberia | 478,085 | 0.35% |
| **Buryats** | **Mongolic** | **Tibetan Buddhism** | **Siberia** | **461,389** | **0.34%** |
| Ingush | Northeast Caucasian | Sunni Islam | Caucasus | 444,833 | 0.32% |
| Germans | Indo-European | Protestant Christianity | European Russia | 394,138 | 0.29% |
| Uzbeks | Turkic | Sunni Islam | Central Asia | 289,862 | 0.21% |
| Tuvans | Turkic | Tibetan Buddhism | Siberia | 263,934 | 0.19% |
| Komi | Uralic | Eastern Orthodox Christianity | European Russia | 228,235 | 0.17% |
| Roma | Indo-European | Eastern Orthodox Christianity | European Russia | 204,958 | 0.15% |
| Tajiks | Indo-European | Sunni Islam | Central Asia | 200,666 | 0.15% |
| Kalmyks | Mongolic | Tibetan Buddhism | Caucasus | 183,372 | 0.13% |
| Georgians | South Caucasian | Eastern Orthodox Christianity | Caucasus | 157,803 | 0.11% |
| Jews | Semitic | Judaism | European Russia | 156,801 | 0.11% |
| Moldovans | Indo-European | Eastern Orthodox Christianity | European Russia | 156,400 | 0.11% |
| Koreans | Korean | Irreligion | Siberia | 153,156 | 0.11% |
| Turks | Turkic | Sunni Islam | Caucasus | 105,058 | 0.08% |
| Kyrgyz | Turkic | Sunni Islam | Central Asia | 103,422 | 0.08% |
| Greeks | Indo-European | Eastern Orthodox Christianity | Caucasus | 85,640 | 0.06% |
| Poles | Indo-European | Catholic Christianity | European Russia | 47,125 | 0.03% |
| Afro-Russians^a^ | Different | Christianity/Islam | European Russia | ≈ 40,000 | ≈ 0.03% |
| Turkmens | Turkic | Sunni Islam | Central Asia | 36,885 | 0.03% |
| Lithuanians | Indo-European | Catholic Christianity | European Russia | 31,377 | 0.02% |
| Khanty | Uralic | Eastern Orthodox Christianity | Siberia | 30,943 | 0.02% |
| **Chinese** | **Sino-Tibetan** | **Chinese folk religion** | **Siberia** | **28,943** | **0.02%** |
| Bulgarians | Indo-European | Eastern Orthodox Christianity | European Russia | 24,038 | 0.02% |
| Finns | Uralic | Protestant Christianity | European Russia | 20,267 | 0.01% |
| Latvians | Indo-European | Protestant Christianity | European Russia | 18,979 | 0.01% |
| Estonians | Uralic | Protestant Christianity | European Russia | 17,875 | 0.01% |
| Chukchi | Chukotko-Kamchatkan | Shamanism | Siberia | 15,908 | 0.01% |
| Vietnamese | Vietnamese | Vietnamese folk religion | European Russia | 13,954 | 0.01% |
| Arabs | Semitic | Sunni Islam | European Russia | 9,583 | 0.01% |
| Indians | Indo-European | Hinduism | European Russia | 4,058 | < 0.01% |
| Persians | Indo-European | Shia Islam | Caucasus | 3,696 | < 0.01% |
| Serbs | Indo-European | Eastern Orthodox Christianity | European Russia | 3,510 | < 0.01% |
| Hungarians | Uralic | Catholic Christianity | European Russia | 2,781 | < 0.01% |
| Americans | Indo-European | Protestant Christianity | European Russia | 1,572 | < 0.01% |
| French | Indo-European | Catholic Christianity | European Russia | 1,475 | < 0.01% |
| Italians | Indo-European | Catholic Christianity | European Russia | 1,370 | < 0.01% |
| Spanish | Indo-European | Catholic Christianity | European Russia | 1,162 | < 0.01% |
| Japanese | Japanese | Mahayana Buddhism | Siberia | 835 | < 0.01% |
| Slovaks | Indo-European | Catholic Christianity | European Russia | 324 | < 0.01% |
| *Note:* Bold entries indicate groups selected for Study 2 (see text).  ^a^ *Source:* O'Flynn, Kevin (26 August 2009). "Russia’s Black Community". The Washington Post. Retrieved 25 February 2010. | | | | | |

*Table S2. The Number of Immigrants by Origin*

| Region | Number | % |
| --- | --- | --- |
| Uzbekistan | 4,082,377 | 26.0% |
| Tajikistan | 2,106,256 | 13.4% |
| Ukraine | 1,792,247 | 11.4% |
| **China** | **1,506,110** | **9.6%** |
| Kyrgyzstan | 879,575 | 5.6% |
| **Armenia** | **657,648** | **4.2%** |
| Azerbaijan | 633,765 | 4.0% |
| Kazakhstan | 587,728 | 3.7% |
| Moldova | 542,004 | 3.5% |
| **Belarus** | **394,449** | **2.5%** |
| Germany | 228,228 | 1.5% |
| South Korea | 193,140 | 1.2% |
| France | 111,409 | 0.7% |
| Arabic countries | ≈ 110,000 | ≈ 0.7% |
| Finland | 106,749 | 0.7% |
| Italy | 103,480 | 0.7% |
| USA | 92,436 | 0.6% |
| India | 84,796 | 0.5% |
| Japan | 75,148 | 0.5% |
| Turkmenistan | 74,225 | 0.5% |
| Vietnam | 72,644 | 0.5% |
| Turkey | 71,717 | 0.5% |
| Iran | 69,395 | 0.4% |
| United Kingdom | 64,345 | 0.4% |
| North Korea | 60,679 | 0.4% |
| Spain | 55,911 | 0.4% |
| Estonia | 55,117 | 0.4% |
| Israel | 52,477 | 0.3% |
| Georgia | 47,687 | 0.3% |
| Latvia | 46,440 | 0.3% |
| Serbia | 41,820 | 0.3% |
| Poland | 37,426 | 0.2% |
| African countries | ≈ 35,000 | ≈ 0.2% |
| Lithuania | 29,252 | 0.2% |
| Argentina | 23,557 | 0.2% |
| Brazil | 22,285 | 0.1% |
| Bulgaria | 14,748 | < 0.1% |
| Greece | 14,106 | < 0.1% |
| Mexico | 13,916 | < 0.1% |
| Slovakia | 12,267 | < 0.1% |
| Hungary | 11,577 | < 0.1% |
| *Note:* Bold entries indicate groups selected for Study 2 (see text).  *Source:* Main Directorate on Issues of Migration, Ministry of Internal Affairs of Russia for 2017. | | |

*Table S3. Descriptive Statistics including Means, Reliability Coefficients, and Cluster Membership*

| Group | *M* (*SD*) | | Difference | Difficulties | Finn | | α | | Cluster |
| --- | --- | --- | --- | --- | --- | --- | --- | --- | --- |
|  | C | W |  |  | C | W | C | W |  |
| Jews | 4.07 (1.05) | 2.04 (1.11) | 2.03* | 7 | .705 | .793 | .820 | .888 | HC-LW |
| Japanese | 4.34 (0.92) | 2.62 (0.95) | 1.71* | 9 | .789 | .627 | .833 | .724 | HC-LW |
| Germans | 4.38 (0.80) | 2.95 (0.95) | 1.43* | 4 | .796 | .637 | .786 | .732 | HC-LW |
| **Chinese** | **4.00 (0.90)** | **2.62 (0.96)** | **1.38*** | **7** | **.771** | **.777** | **.813** | **.839** | **HC-LW** |
| English | 3.42 (1.14) | 2.11 (0.95) | 1.31* | 12 | .741 | .756 | .867 | .820 | HC-LW |
| Americans | 3.71 (1.03) | 2.54 (1.08) | 1.18* | 11 | .781 | .757 | .863 | .860 | HC-LW |
| Finns | 4.10 (0.87) | 3.26 (0.89) | 0.84* | 16 | .866 | .762 | .882 | .800 | HC-LW |
| Koreans | 3.86 (0.97) | 3.06 (0.94) | 0.80* | 15 | .769 | .703 | .836 | .778 | HC-LW |
| French | 3.90 (0.85) | 3.14 (0.96) | 0.76* | 16 | .824 | .777 | .839 | .837 | HC-LW |
| Poles | 3.09 (1.09) | 2.42 (1.04) | 0.67* | 12 | .802 | .774 | .889 | .861 | LC-LW |
| Arabs | 2.93 (1.20) | 2.29 (1.02) | 0.64* | 18 | .731 | .710 | .875 | .813 | LC-LW |
| Turks | 2.69 (1.02) | 2.12 (0.81) | 0.57* | 14 | .774 | .701 | .856 | .698 | LC-LW |
| Lithuanians | 2.97 (1.10) | 2.41 (1.06) | 0.57* | 27 | .776 | .806 | .877 | .884 | LC-LW |
| Estonians | 2.97 (1.13) | 2.41 (1.05) | 0.56* | 29 | .802 | .772 | .897 | .863 | LC-LW |
| Latvians | 2.76 (1.08) | 2.28 (1.12) | 0.48* | 19 | .772 | .813 | .869 | .901 | LC-LW |
| Hungarians | 3.27 (0.88) | 2.94 (0.88) | 0.33* | 57 | .803 | .826 | .829 | .850 | MC-MW |
| Tatars | 3.47 (0.97) | 3.15 (0.97) | 0.32* | 8 | .837 | .820 | .883 | .872 | MC-MW |
| Italians | 3.69 (0.89) | 3.38 (1.00) | 0.31* | 10 | .785 | .712 | .818 | .807 | MC-MW |
| Spanish | 3.22 (0.89) | 2.94 (0.90) | 0.28* | 36 | .773 | .751 | .808 | .796 | MC-MW |
| Ukrainians | 3.02 (1.02) | 2.85 (1.13) | 0.17* | 9 | .763 | .764 | .848 | .877 | MC-MW |
| Persians | 2.90 (1.00) | 2.73 (0.98) | 0.17* | 45 | .841 | .820 | .894 | .875 | MC-MW |
| Roma | 1.84 (0.96) | 1.68 (0.90) | 0.16* | 12 | .722 | .801 | .801 | .835 | LC-LW |
| **Armenians** | **3.60 (1.03)** | **3.45 (1.12)** | **0.15*** | **3** | **.780** | **.745** | **.861** | **.865** | **MC-MW** |
| Slovaks | 3.29 (0.94) | 3.17 (1.04) | 0.11 | 34 | .762 | .792 | .822 | .872 | MC-MW |
| Argentines | 3.35 (0.90) | 3.24 (1.03) | 0.11 | 59 | .730 | .715 | .775 | .819 | MC-MW |
| Azerbaijanis | 2.66 (0.98) | 2.56 (0.96) | 0.10 | 8 | .765 | .729 | .837 | .805 | LC-LW |
| Vietnamese | 3.12 (0.94) | 3.05 (0.97) | 0.07 | 32 | .684 | .721 | .763 | .803 | MC-MW |
| **Chechens** | **1.96 (1.03)** | **1.93 (1.01)** | **0.03** | **3** | **.833** | **.723** | **.895** | **.818** | **LC-LW** |
| Indians | 2.81 (1.02) | 2.79 (1.04) | 0.02 | 32 | .739 | .707 | .833 | .821 | MC-MW |
| Tajiks | 2.76 (1.11) | 2.76 (1.11) | 0.00 | 10 | .765 | .770 | .873 | .874 | LC-LW |
| Avars | 2.78 (1.22) | 2.81 (1.29) | -0.03 | 27 | .818 | .775 | .919 | .910 | MC-MW |
| Kazakhs | 3.43 (1.06) | 3.47 (1.10) | -0.04 | 9 | .827 | .785 | .899 | .881 | MC-MW |
| Turkmens | 2.44 (0.98) | 2.47 (0.92) | -0.04 | 26 | .797 | .799 | .858 | .843 | LC-LW |
| Moldovans | 2.88 (0.94) | 2.92 (0.98) | -0.04 | 21 | .749 | .710 | .812 | .799 | MC-MW |
| Kyrgyz | 2.88 (1.04) | 2.92 (1.09) | -0.05 | 17 | .824 | .813 | .891 | .896 | MC-MW |
| Russians | 4.31 (0.69) | 4.37 (0.72) | -0.07 | 4 | .876 | .861 | .828 | .820 | HC-HW |
| Uzbeks | 2.70 (0.98) | 2.77 (1.02) | -0.07 | 11 | .734 | .739 | .814 | .831 | LC-LW |
| Brazilians | 2.98 (0.76) | 3.07 (0.86) | -0.09 | 44 | .742 | .745 | .704 | .771 | MC-MW |
| Ingush | 2.78 (0.99) | 2.87 (1.06) | -0.09 | 45 | .761 | .763 | .836 | .860 | MC-MW |
| Bashkirs | 3.29 (0.92) | 3.39 (0.96) | -0.10 | 18 | .778 | .776 | .825 | .838 | MC-MW |
| Kabardians | 3.07 (1.07) | 3.18 (1.12) | -0.11 | 34 | .824 | .800 | .898 | .894 | MC-MW |
| Ossetians | 3.10 (1.05) | 3.22 (1.14) | -0.12 | 20 | .833 | .737 | .899 | .865 | MC-MW |
| Mordvins | 3.01 (0.89) | 3.17 (0.88) | -0.16* | 40 | .831 | .815 | .857 | .842 | MC-MW |
| Chuvashs | 3.04 (0.92) | 3.21 (0.96) | -0.17* | 49 | .770 | .740 | .819 | .813 | MC-MW |
| Serbs | 3.57 (0.94) | 3.74 (1.09) | -0.17* | 28 | .785 | .753 | .836 | .862 | HC-HW |
| Georgians | 3.09 (1.00) | 3.26 (1.05) | -0.18* | 7 | .754 | .757 | .836 | .852 | MC-MW |
| Bulgarians | 3.20 (0.80) | 3.37 (0.93) | -0.18* | 24 | .818 | .812 | .812 | .856 | MC-MW |
| Greeks | 3.29 (0.86) | 3.47 (0.85) | -0.18* | 31 | .801 | .806 | .820 | .822 | MC-MW |
| Mexicans | 2.81 (0.85) | 3.00 (0.88) | -0.19* | 39 | .820 | .752 | .835 | .785 | MC-MW |
| Tuvans | 2.93 (0.98) | 3.13 (1.08) | -0.20* | 66 | .765 | .771 | .836 | .869 | MC-MW |
| Khanty | 3.13 (0.98) | 3.34 (1.01) | -0.21* | 46 | .763 | .757 | .836 | .840 | MC-MW |
| Dargins | 2.08 (1.02) | 2.29 (1.10) | -0.21* | 10 | .830 | .787 | .891 | .882 | LC-LW |
| Kalmyks | 2.82 (0.91) | 3.03 (0.93) | -0.21* | 42 | .810 | .812 | .847 | .854 | MC-MW |
| **Belarusians** | **3.98 (0.83)** | **4.20 (0.83)** | **-0.22*** | **7** | **.837** | **.854** | **.844** | **.858** | **HC-HW** |
| Komi | 2.98 (0.90) | 3.25 (0.87) | -0.27* | 52 | .808 | .806 | .841 | .830 | MC-MW |
| Yakuts | 3.28 (0.94) | 3.60 (0.95) | -0.32* | 25 | .725 | .778 | .792 | .838 | MC-MW |
| Udmurts | 3.06 (0.93) | 3.39 (0.94) | -0.33* | 58 | .781 | .719 | .831 | .789 | MC-MW |
| **Buryats** | **2.84 (0.89)** | **3.28 (0.94)** | **-0.44*** | **34** | **.793** | **.838** | **.825** | **.877** | **MC-MW** |
| Africans | 2.17 (0.98) | 2.67 (0.97) | -0.50* | 30 | .808 | .720 | .866 | .802 | LC-LW |
| Chukchi | 2.79 (0.97) | 3.54 (0.96) | -0.75* | 42 | .746 | .657 | .819 | .752 | MC-MW |
| *Note.* C = competence; W = warmth; Difference = difference between competence-warmth dimensions; Difficulties = number of mentions of a group being difficult for providing an opinion; Finn = Finn's coefficient of interrater agreement on the group’s ratings; α = Cronbach's alpha showing internal consistency of three-item scale for that trait for that group. **Bold** groups appear in Study 2. Cluster membership was determined by cluster analysis.  * *p* < .05; the mean difference equal or more then |0.50| provides medium effect size (*d* = 0.50). | | | | | | | | | |
