# Supplementary material for: Mapping Ethnic Stereotypes and Their Antecedents in Russia: The Stereotype Content Model
Source: Front Psychol. 2019 Jul 16;10:1643. doi: 10.3389/fpsyg.2019.01643 (PMC6646730; doi:10.3389/fpsyg.2019.01643)

Figure S1. Network graph and centrality plot for Belarusians (HC-HW)


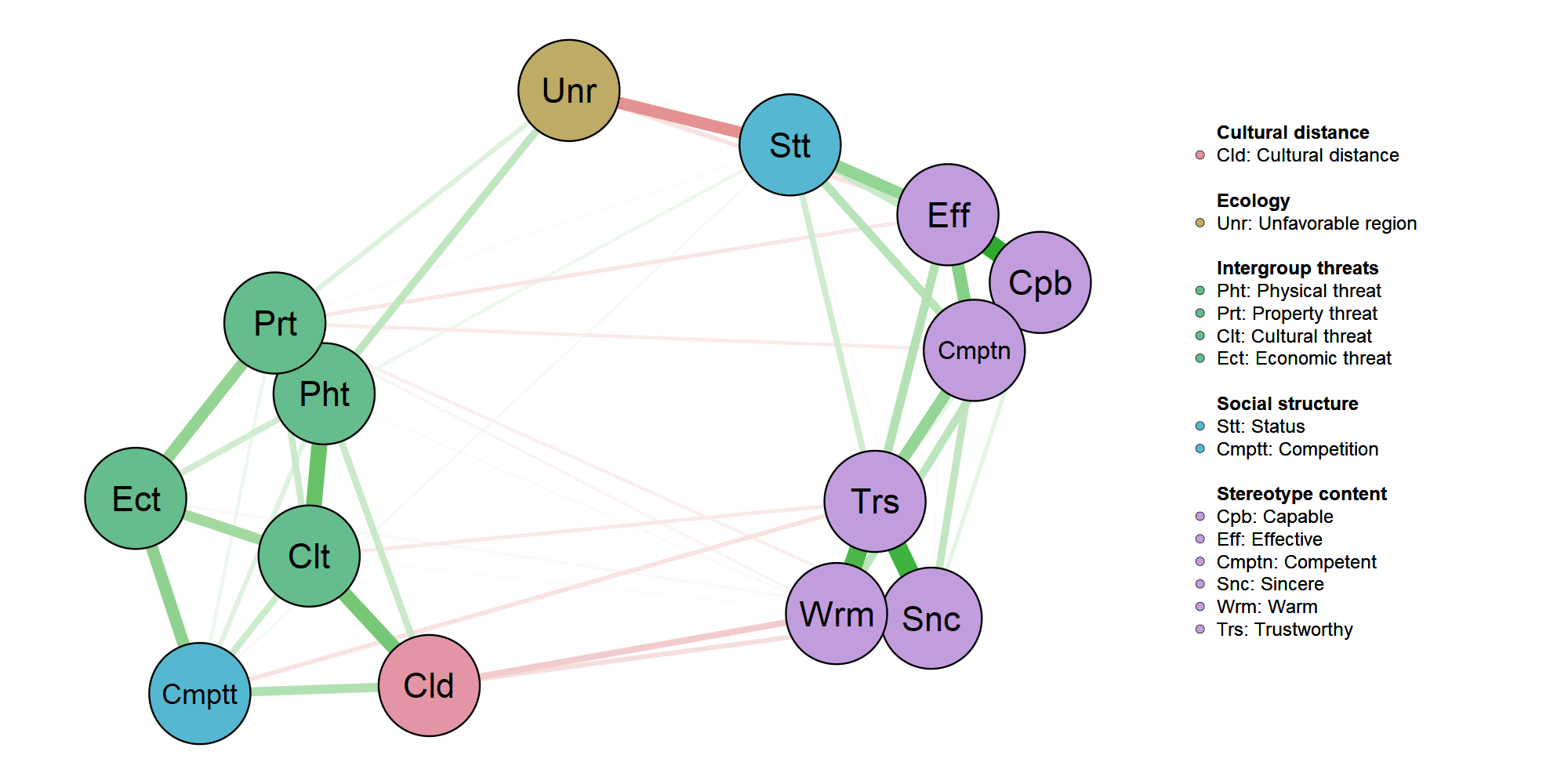


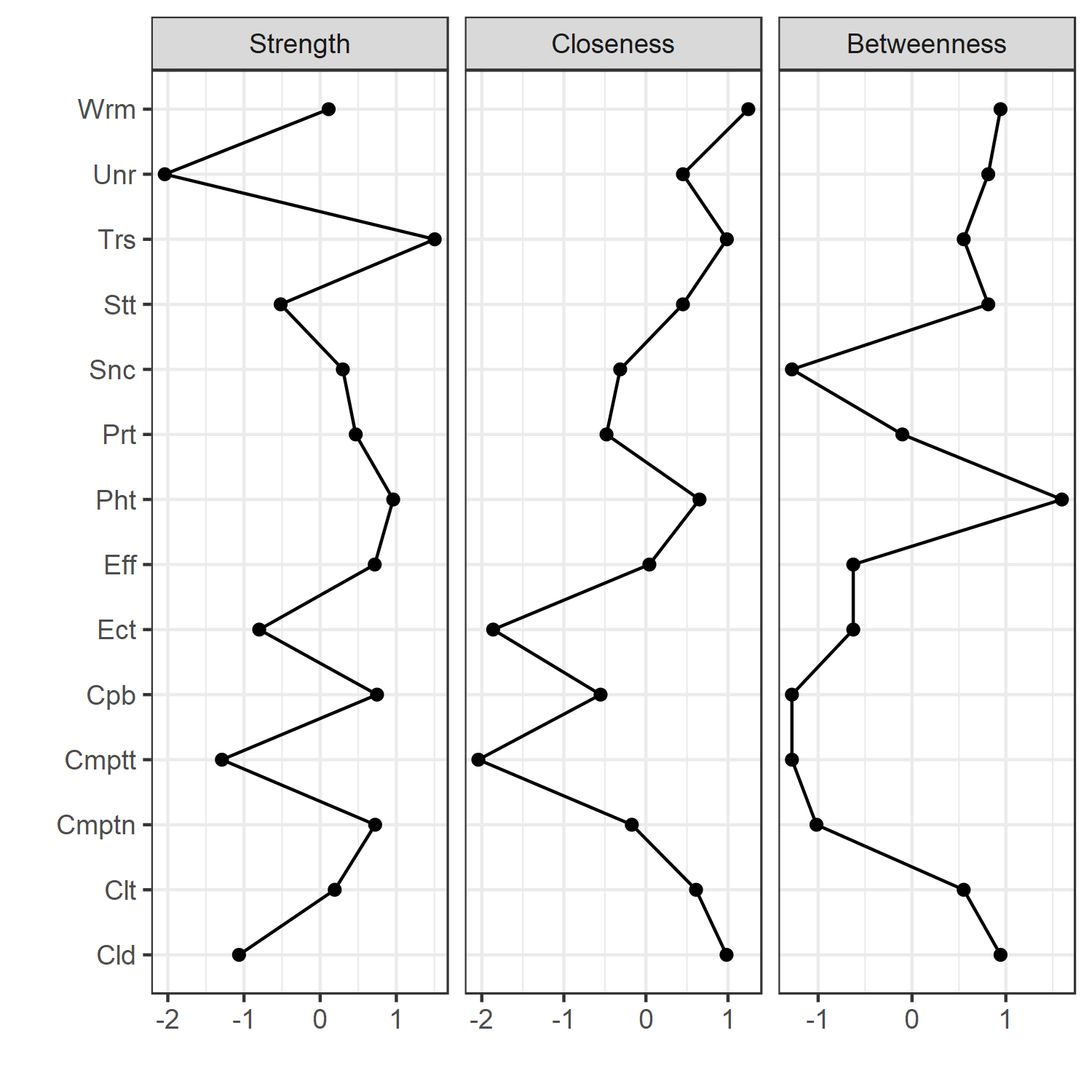


Figure S2. Network graph and centrality plot for Armenians (MC-MW)


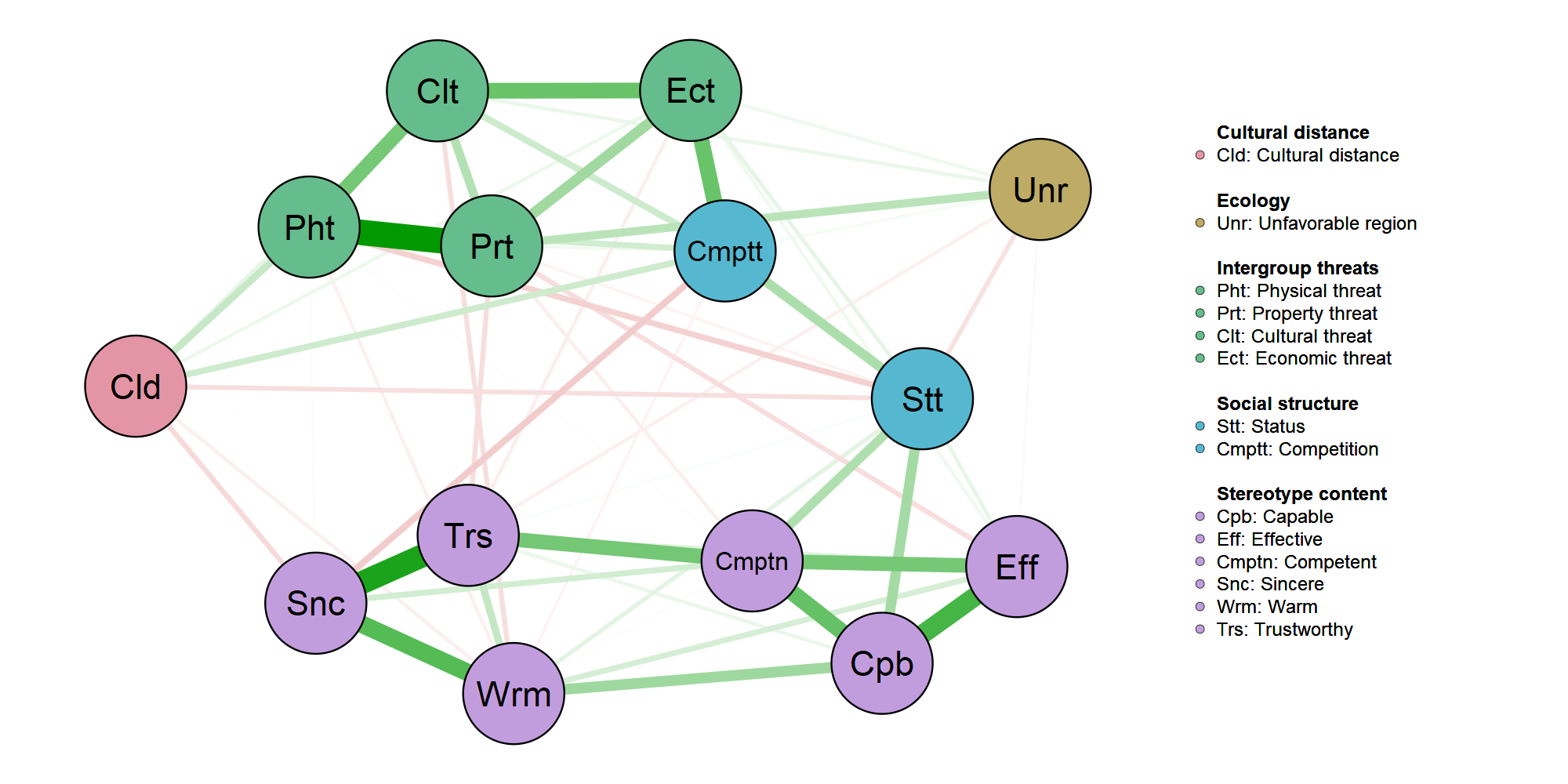


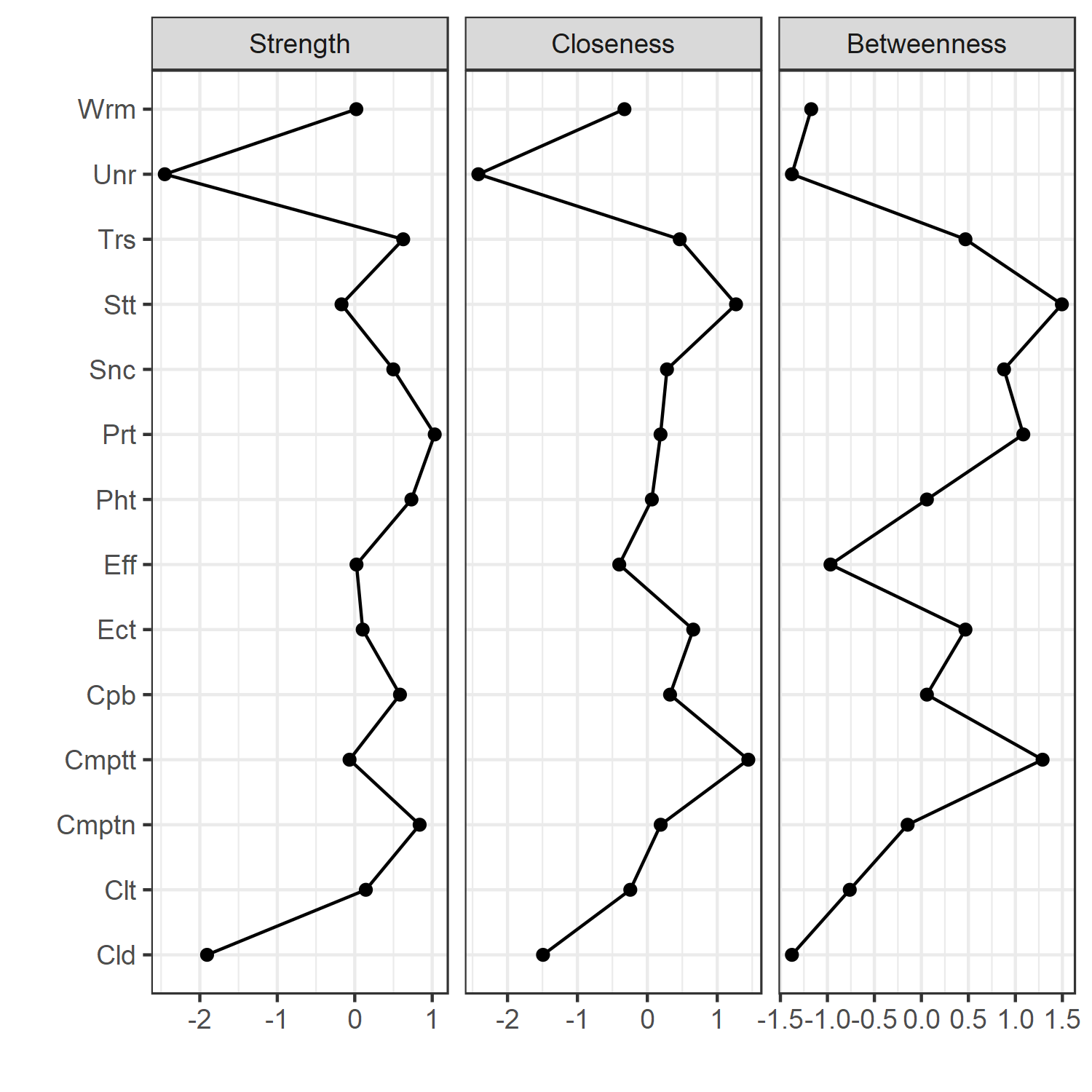


Figure S3. Network graph and centrality plot for Buryats (LC-HW)


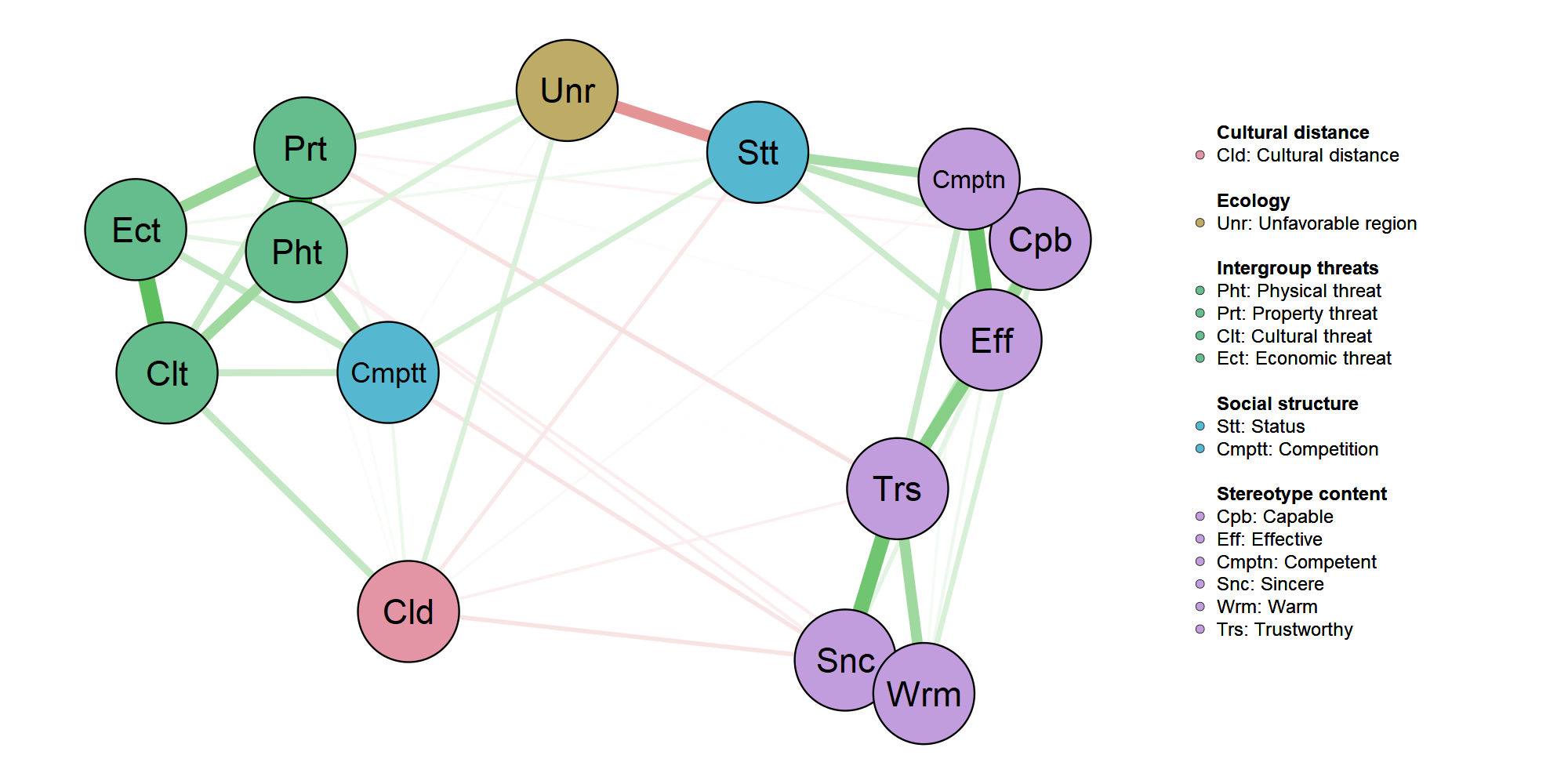


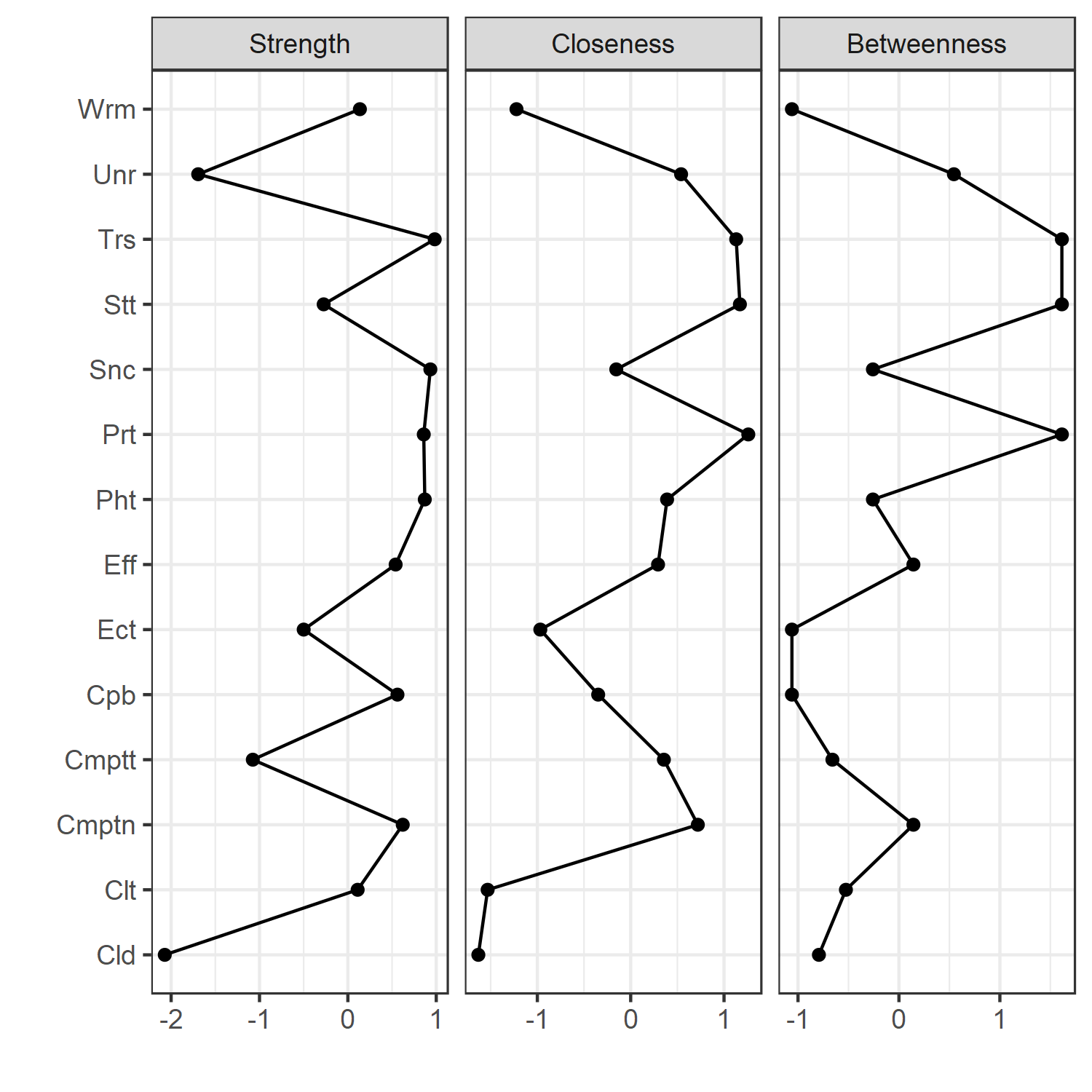


Figure S4. Network graph and centrality plot for Chechens (LC-LW)


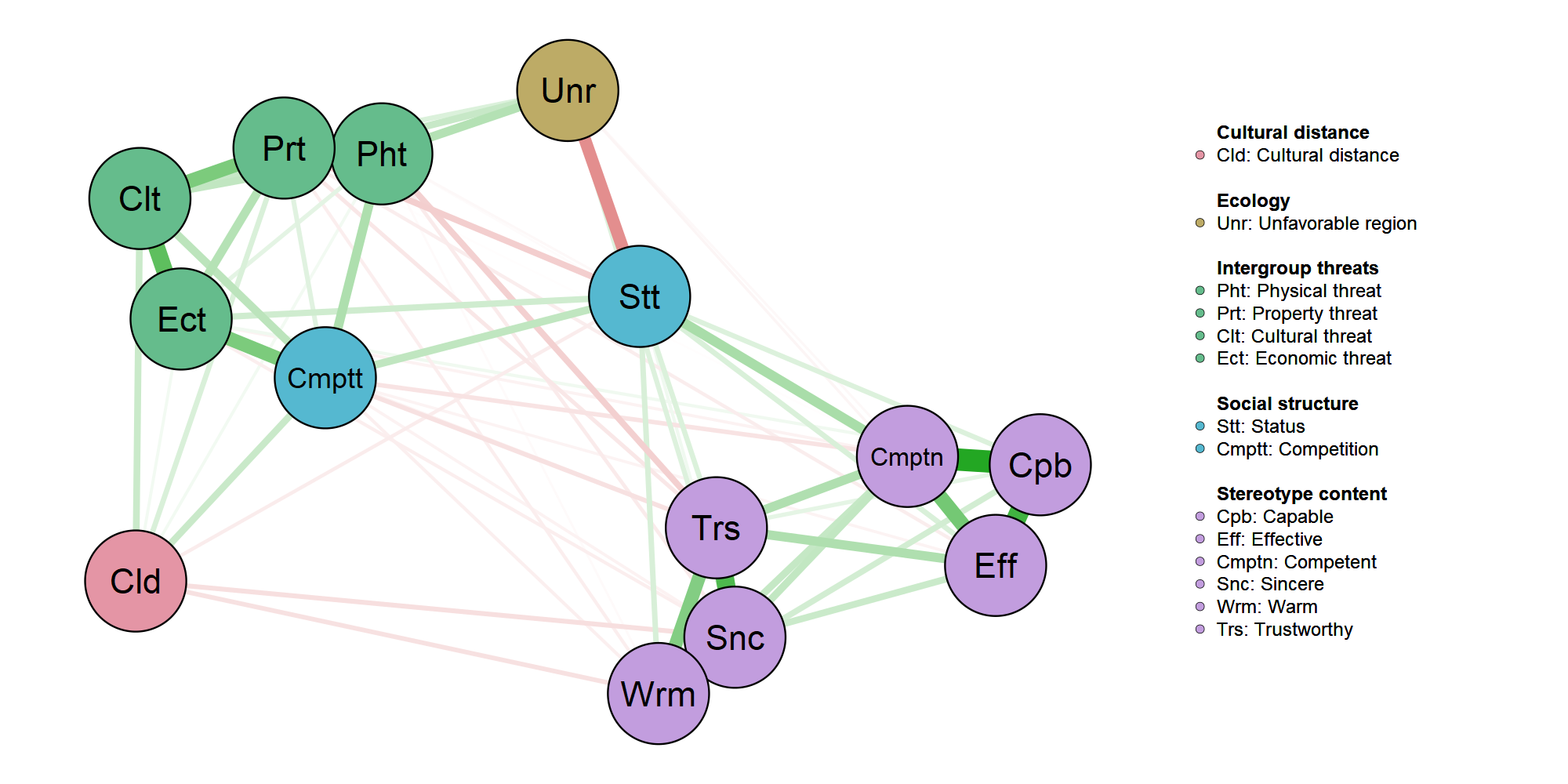


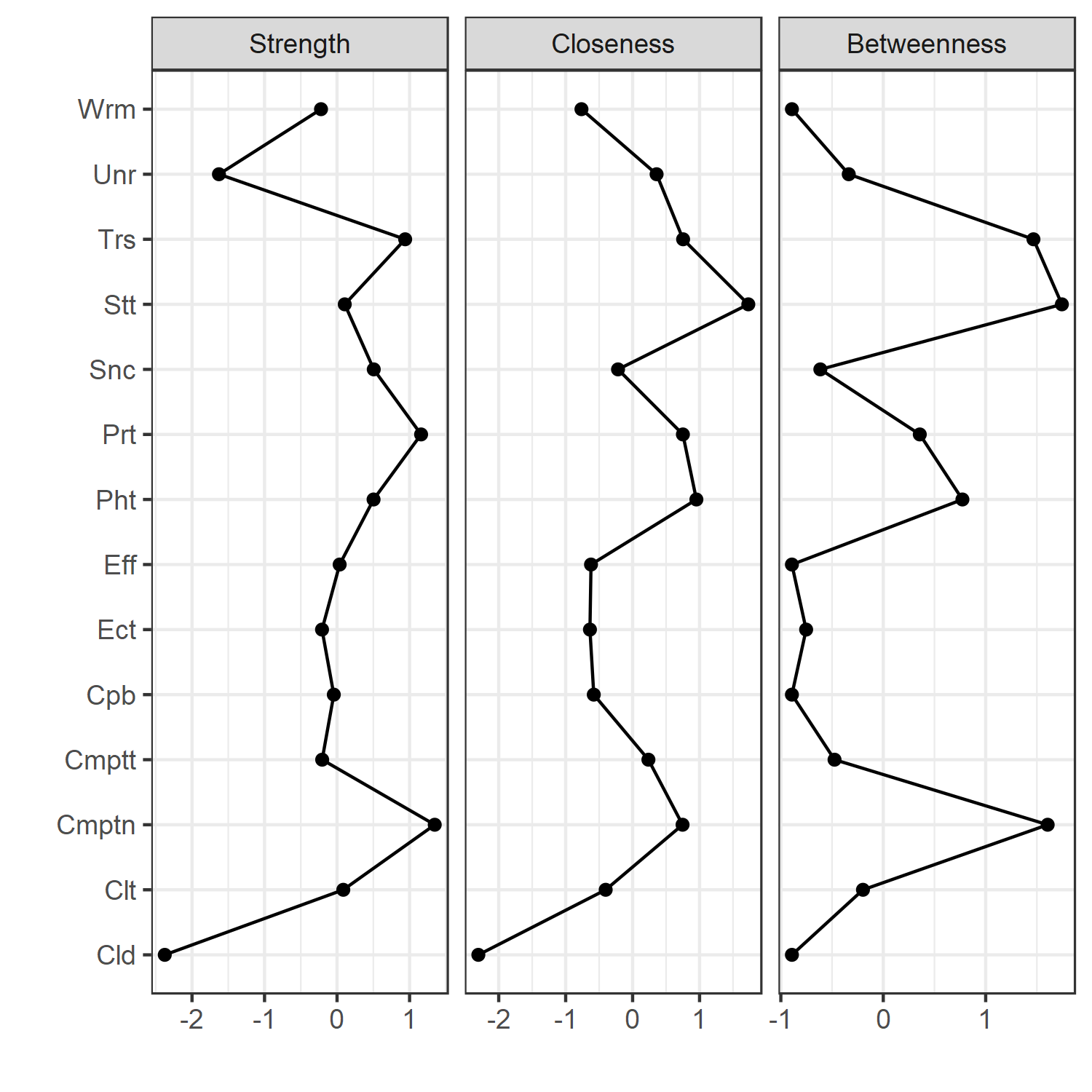


Figure S5. Network graph and centrality plot for Chinese (HC-LW)


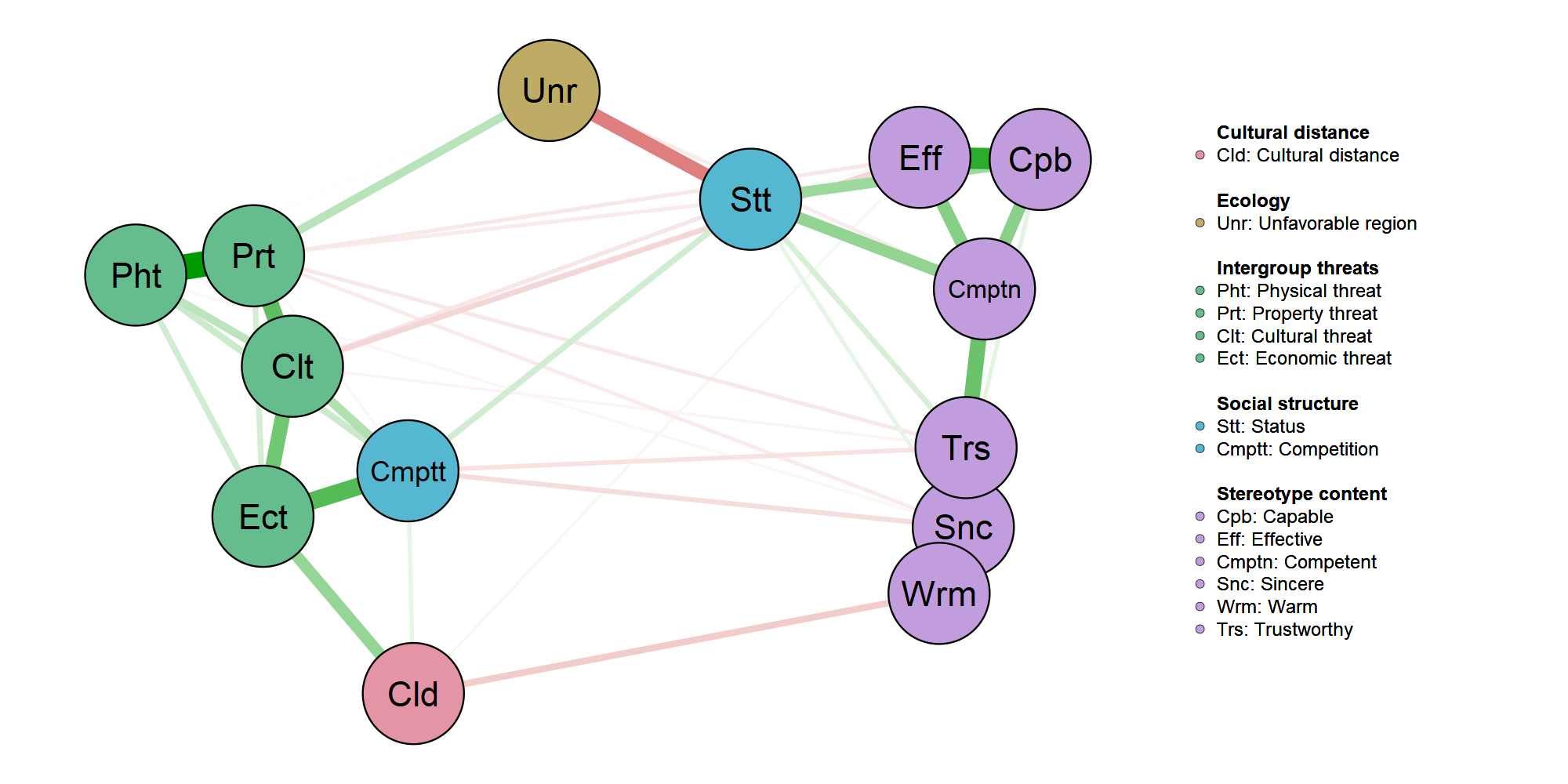


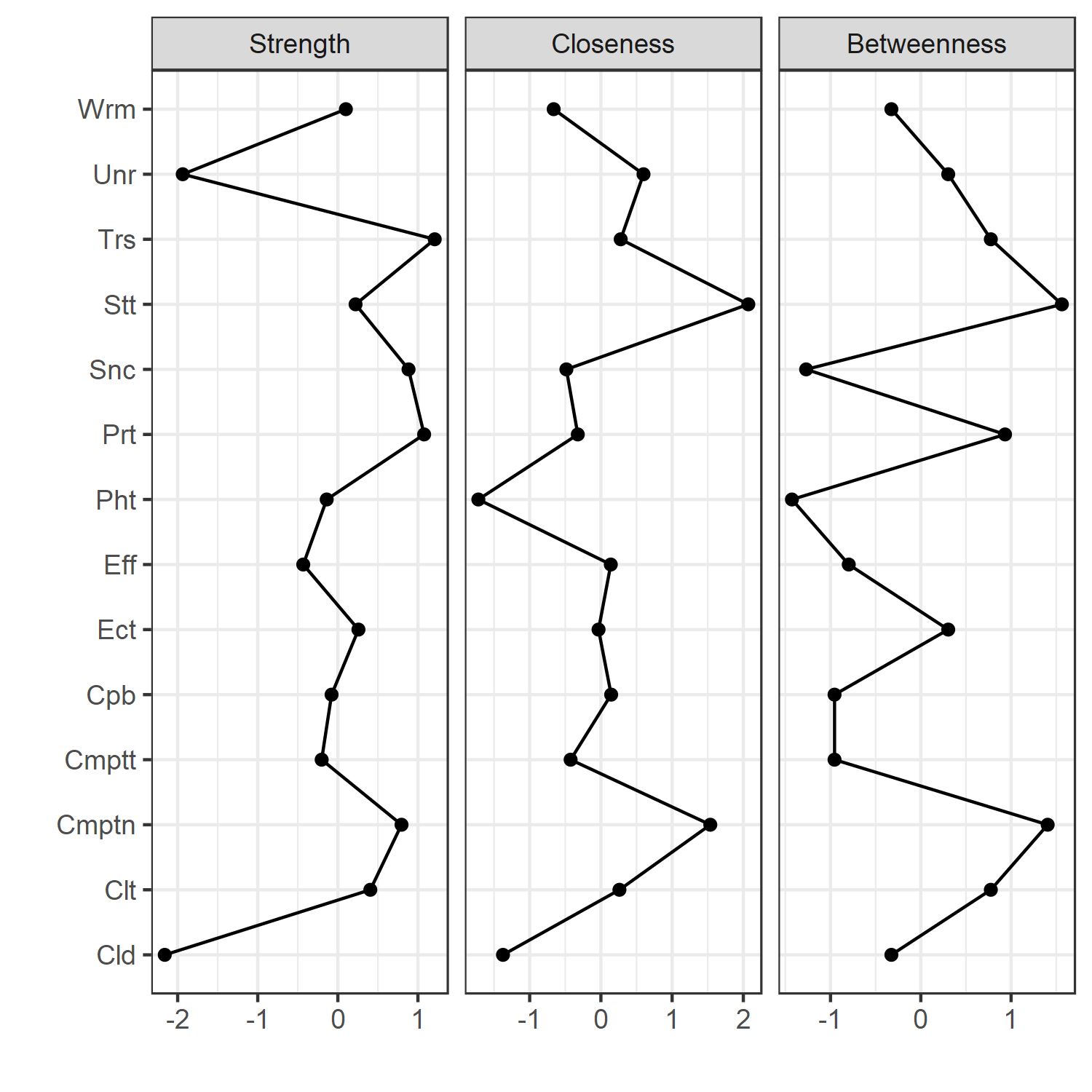

Supplement: Supplementary file 2 [file Table_2.docx]
